# Distinguishing African bovids using Zooarchaeology by Mass Spectrometry (ZooMS): New peptide markers and insights into Iron Age economies in Zambia

**DOI:** 10.1371/journal.pone.0251061

**Published:** 2021-05-18

**Authors:** Anneke Janzen, Kristine Korzow Richter, Ogeto Mwebi, Samantha Brown, Veronicah Onduso, Filia Gatwiri, Emmanuel Ndiema, Maggie Katongo, Steven T. Goldstein, Katerina Douka, Nicole Boivin

**Affiliations:** 1 Department of Archaeology, Max-Planck Institute for the Science of Human History, Jena, Germany; 2 Department of Anthropology, University of Tennessee, Knoxville, Tennessee, United States of America; 3 Department of Anthropology, Harvard University, Boston, Massachusetts, United States of America; 4 Department of Zoology, Osteology Section, National Museums of Kenya, Nairobi, Kenya; 5 Department of Earth Sciences, Archaeology Section, National Museums of Kenya, Nairobi, Kenya; 6 Department of Archaeology, Livingstone Museum, Livingstone, Zambia; 7 School of Social Science, The University of Queensland, Brisbane, Australia; 8 Department of Anthropology and Archaeology, University of Calgary, Calgary, Canada; 9 Department of Anthropology, National Museum of Natural History, Smithsonian Institution, Washington, D.C., United States of America; Monash University, AUSTRALIA

## Abstract

Assessing past foodways, subsistence strategies, and environments depends on the accurate identification of animals in the archaeological record. The high rates of fragmentation and often poor preservation of animal bones at many archaeological sites across sub-Saharan Africa have rendered archaeofaunal specimens unidentifiable beyond broad categories, such as “large mammal” or “medium bovid”. Identification of archaeofaunal specimens through Zooarchaeology by Mass Spectrometry (ZooMS), or peptide mass fingerprinting of bone collagen, offers an avenue for identification of morphologically ambiguous or unidentifiable bone fragments from such assemblages. However, application of ZooMS analysis has been hindered by a lack of complete reference peptide markers for African taxa, particularly bovids. Here we present the complete set of confirmed ZooMS peptide markers for members of all African bovid tribes. We also identify two novel peptide markers that can be used to further distinguish between bovid groups. We demonstrate that nearly all African bovid subfamilies are distinguishable using ZooMS methods, and some differences exist between tribes or sub-tribes, as is the case for Bovina (cattle) vs. Bubalina (African buffalo) within the subfamily Bovinae. We use ZooMS analysis to identify specimens from extremely fragmented faunal assemblages from six Late Holocene archaeological sites in Zambia. ZooMS-based identifications reveal greater taxonomic richness than analyses based solely on morphology, and these new identifications illuminate Iron Age subsistence economies c. 2200–500 cal BP. While the Iron Age in Zambia is associated with the transition from hunting and foraging to the development of farming and herding, our results demonstrate the continued reliance on wild bovids among Iron Age communities in central and southwestern Zambia Iron Age and herding focused primarily on cattle. We also outline further potential applications of ZooMS in African archaeology.

## Introduction

**Fig 1:**
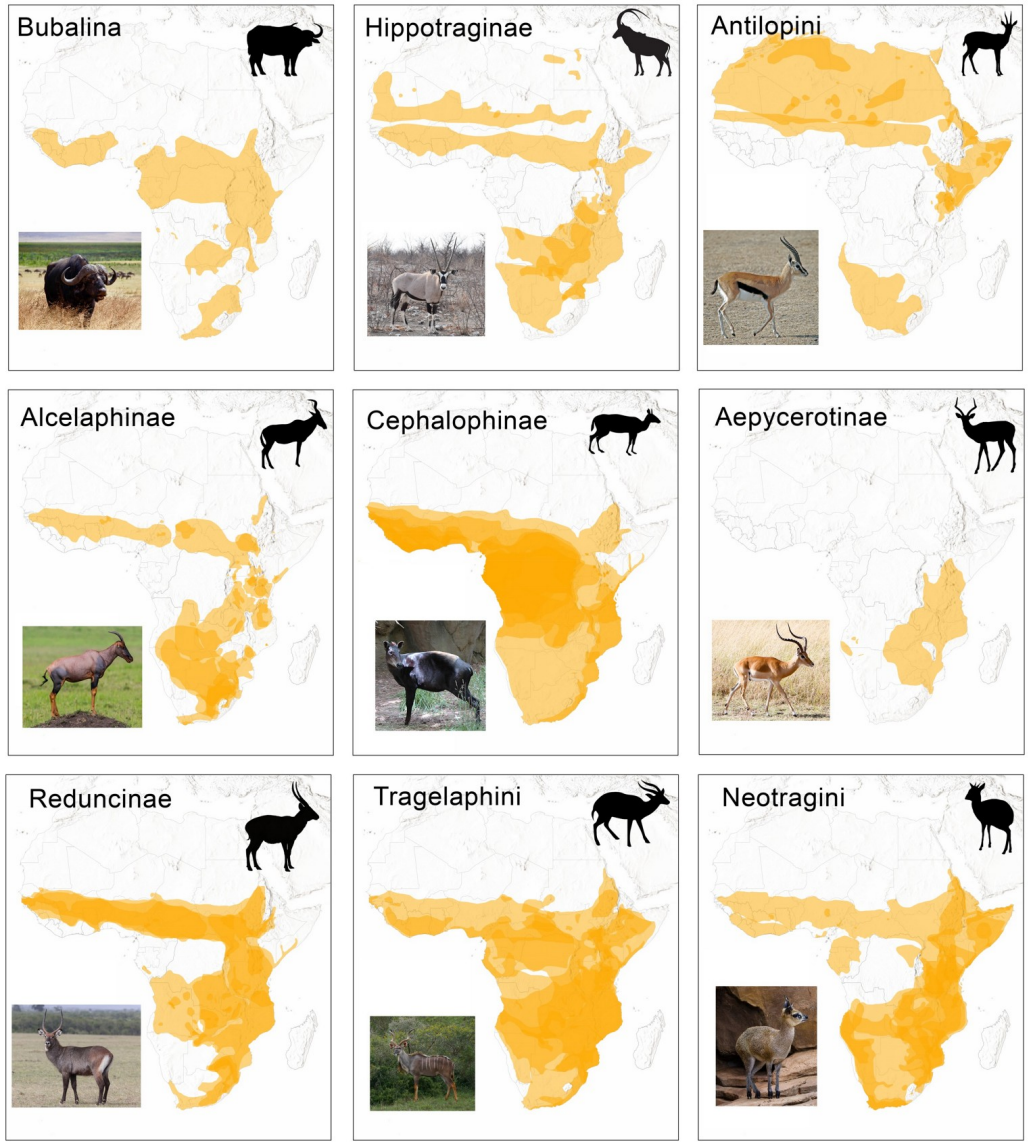
Distribution maps of African species belonging to each of the wild bovid subfamilies, tribes, or sub-tribes analyzed in this study. Distribution data from IUCN [16]. Basemap from [17].

**Fig 2:**
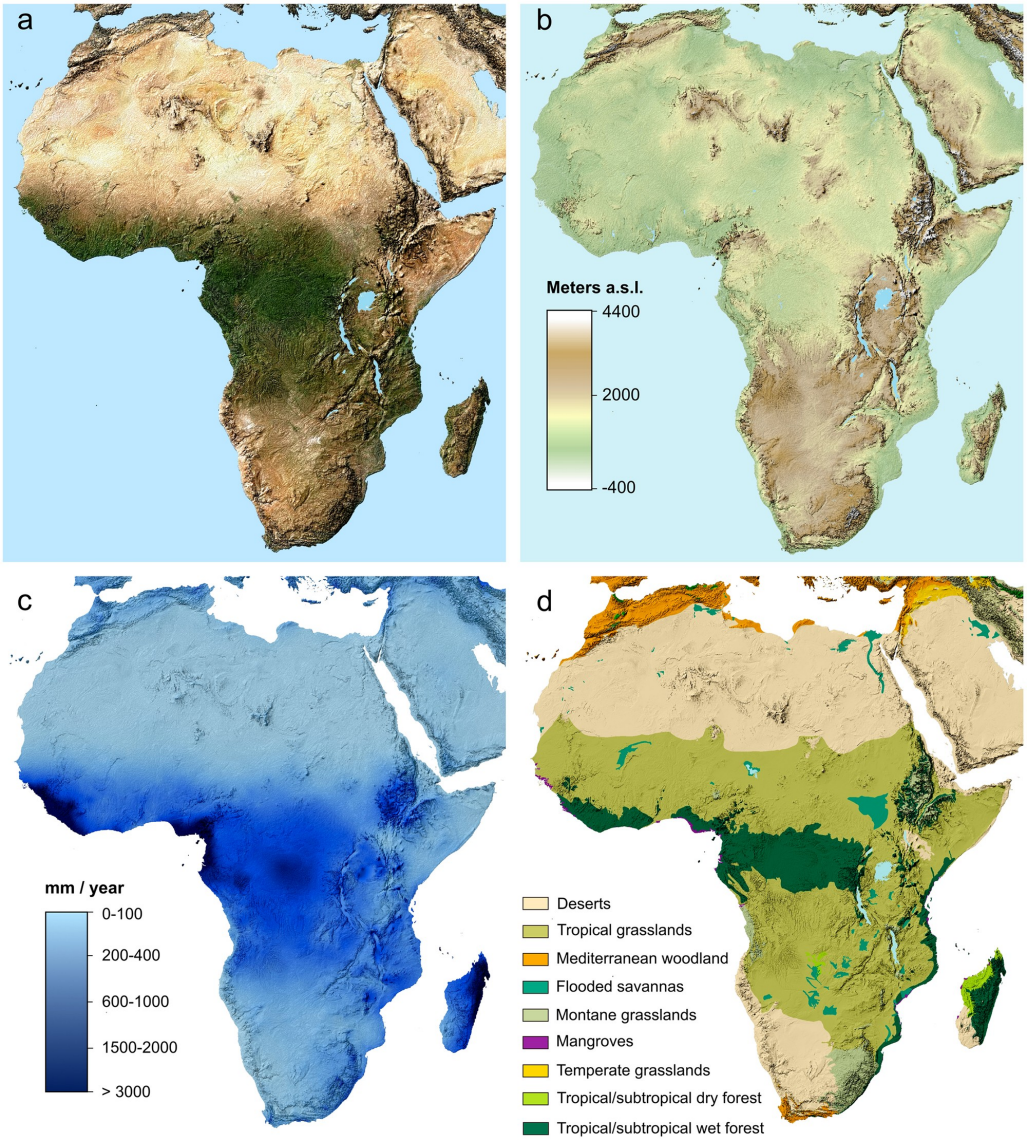
(A) satellite imagery [18]; (B) elevation (meters above sea level) [19]; (C) mean annual precipitation (mm) [20]; (D) ecoregions [21].

**Fig 3:**
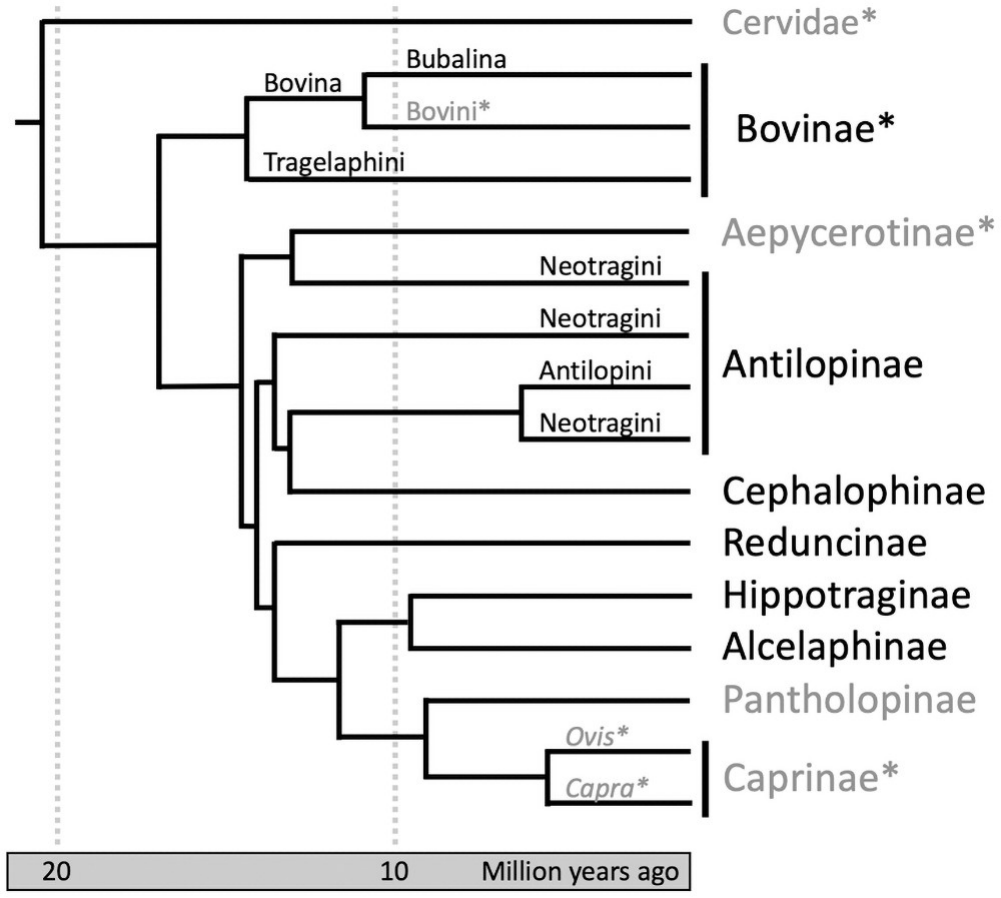
A phylogenetic tree of Bovidae (after Chen et al. 2019). Families with species that have previously published ZooMS markers are indicated with an asterisk (*). Families that we have explored in this study are in black. Antilopinae is shown here as non-monophyletic. The placement of Pantholopinae is disputed with the alternate placement putting the single species as a subfamily within Caprinae.

Secure identification of animal taxa in the archaeological record is critical for assessing past subsistence strategies, environments, foodways, and tracking species dispersals and translocations. A major challenge in exploring these questions, and in analysis of African archaeofaunal assemblages in general, is the sheer number of bovid species in most regions. Globally, the Bovidae family composes over 50% of ungulates with over 140 extant species. Bovids exhibit significant diversity in body size, morphology, geographical range, diet, and behavior (Fig 1), and inhabit a range of environments across Africa, which vary greatly in terms of topography, climate, and vegetation (Fig 2). Sub-Saharan Africa is home to the greatest diversity of bovids on Earth, including species from eight of the nine subfamilies of Bovidae (Figs 1 and 3). Three domestic bovid species are also widespread across the continent: cattle (*Bos taurus* and *Bos indicus*), sheep (*Ovis aries*), and goat (*Capra hircus*). Given the considerable richness of bovid species across much of the African continent, these morphologically similar taxa can be difficult to distinguish when fragmentary. In the absence of robust, diagnostic elements or teeth, highly fragmented assemblages are often characterized by mostly coarse identifications to the family level and body size class (e.g. Bovid 2) [1]. A range of processes contribute to fragmentation as well as deletion of identifiable bone ends, including culinary processing [2, 3], carnivore damage [4–6], and density-mediated attrition, in which less-dense elements or element portions fail to survive taphonomic processes [7, 8]. Manufacture of bone tools or objects also typically involves removal of epiphyses and obliterates identifiable features [9] Where reported in faunal analyses, the less- or non-identifiable component comprises the bulk of faunal assemblages, and bovids comprise the majority of minimally-identifiable mammal remains [10–15]. Given this large proportion of remains that are not attributable to taxon in so many archaeological sites, zooarchaeologists have been limited in their interpretations of African subsistence strategies, foodways, and the economic and symbolic importance of animals in the past.

These taphonomic issues make it particularly difficult to evaluate taxonomic frequencies of bovid species in sites with high bone fragmentation and poor preservation, hindering assessments of hunting strategies, foodways, past environments, and subsistence economies. In particular, tracking the expansion and nature of food production in Africa is challenging because initial evidence for livestock in a region is often patchy [22–25], and identifications may be hotly debated [26–29]. For example, early evidence for domesticates in Kenya south of the Lake Turkana Basin before 3300 BP are sparse, and limited to a few identifiable elements at a handful of sites, such as in Kansyore levels (c. 4400 to 4000 cal. BP) at Wadh Lang’o [25] and at c. 4100 BP at Enkapune Ya Muto [23]. In southern Africa, early zooarchaeological evidence for livestock is also limited to a few morphologically identifiable specimens. Sites including Toteng 1 [30] and Spoegrivier [31–33] have early evidence for sheep at c. 2070 and 2100 BP, respectively. Efforts in identifying early domesticates through molecular methods such as ancient DNA (aDNA) have been met with controversy [26–29]. Many early herders and farmers relied on domesticates and terrestrial wild species [13, 34–36] and reliance on wild bovids, particularly in forested areas, has continued into recent centuries in many parts of the continent [37]. Despite their centrality in archaeological discussions of African economies and diets, the considerable richness of native bovid species and their gross morphological similarity, as well as their osteological resemblance to domesticates, complicates assessments of their exploitation in the past.

Zooarchaeology by Mass Spectrometry (ZooMS), or peptide mass fingerprinting of bone collagen to taxonomically identify morphological ambiguous mammal, fish, and reptile bone, has been successfully applied in many parts of the world [38–42]. Unlike ancient aDNA analysis, ZooMS derives identifications from the study of bone collagen, which is well-preserved in many archaeological contexts. Given the often poor preservation of aDNA, particularly outside of temperate regions and when petrous bone is unavailable, ZooMS-based studies accordingly have higher success rates than aDNA studies [43]. In East African contexts, ZooMS has proven to be effective in examining translocations of Asian species and identifying domesticates, but in limited contexts [44, 45]. Coutu and colleagues [46] also used ZooMS to investigate the early ivory trade in southern Africa in the 7^th^ century CE. Despite the clear value of ZooMS, however, very few studies have applied the method to study indigenous African taxa, and those that have done so have been focused on worked bone artifacts in limited geographical areas, and have drawn upon a small reference library of African bovid species [47–49]. Two recent studies have explored early evidence for caprines. Le Meillour and colleagues used paleoproteomics on potential caprines to clarify the absence of domestic livestock at Leopard Cave in central Namibia until c. 960 BP [49]. Most recently, Coutu et al [33] identified a new ZooMS marker that separates sheep from wild bovids to explore the introduction of domestic sheep into southern Africa. However, the lack of a comprehensive peptide marker reference library for wild African bovids has greatly limited the applicability of ZooMS in African archaeology. The present study remedies this situation by providing these reference markers.

Here, we present the first extensive study identifying ZooMS peptide markers for all indigenous African bovid tribes, and character two new peptide markers for bovids. This database allows, for the first time, large-scale systematic identifications of otherwise unidentifiable fragments in African archaeofaunal assemblages, as well as rapid confirmation of identifications of domesticate and wild species that lack confidence due to morphological similarity. We apply the newly generated reference African bovid peptide sequences produced in this study to the investigation of extremely fragmented archaeofaunal assemblages from several recently excavated Iron Age (c. 2200–500 cal BP) archaeological sites in central and southwestern Zambia. Today, Zambia is home to members of most of the bovid groups described in this study (Fig 1), and may have included more taxa in the past. Our results reveal that subsistence economies were grounded in cattle-based pastoralism, and included hunting of wild bovids, particularly cephalophines (duikers). We also demonstrate the validity and reliability of ZooMS methods in African contexts and provide a baseline for collagen recovery rates for typical highly fragmented African assemblages. The newly developed African bovid ZooMS reference database offers a novel avenue for addressing key challenges to traditional zooarchaeological analyses, and thus more effectively exploring African hunting and subsistence strategies, foodways, and past environments.

## Faunal identifications and key research questions

While osteologically similar, bovids are incredibly diverse in their diets and the environments they inhabit. For example, extant members of the subfamily Alcelaphinae—wildebeest (*Connochaetes taurinus and Connochaetes gnou*), hartebeest (*Alcelaphus buselaphus*), tsessebe/topi (*Damaliscus lunatus*), and blesbok/bontebok (*Damaliscus pygargus*)—are dedicated grazers that inhabit open grasslands, shrublands, and floodplains [50]. The sole member of the subfamily Aepycerotinae, the impala (*Aepyceros melampus*), is an obligate drinker and mixed feeder, preferring woodland and savannah ecotones [51]. In contrast, members of the subfamily Cephalophinae (e.g. *Sylvicapra grimmia*, *Cephalophus sp*.)—the duikers—are primarily browsers. These smaller-bodied bovids prefer more wooded habitats, though substantial variation in habitat preference exists among species within this group [52]. While variation in diet and habitat preference exists among species within all bovid groups, these broad differences in diet among bovid tribes are apparent in carbon stable isotope data, reflecting differences in the photosynthetic pathways of grasses versus trees and shrubs at low elevations in the tropics [53, 54].

Given their habitat and dietary differences [55, 56], taxonomic identifications of bovids beyond the family level are crucial for inferring past environments [57–63]. Species-level identifications are most effective, allowing for detailed morphological studies that can further clarify environmental context [64, 65], but broad identifications to tribe or subfamily can still provide useful information, particularly in poorly preserved or small assemblages in which few specimens are morphologically identifiable to genus or species. Coarse identifications of bovids to tribe also allow for improved interpretation of stable isotope data. For example, carbon isotope analysis of mixed feeder species (e.g. impala or goat) can clarify the degree of brushy vegetation or grass in an environment. Similarly, identifications of broad taxonomic groups are useful for interpreting δ^18^O values of evaporation-sensitive and -insensitive animals [66–69].

**Fig 4:**
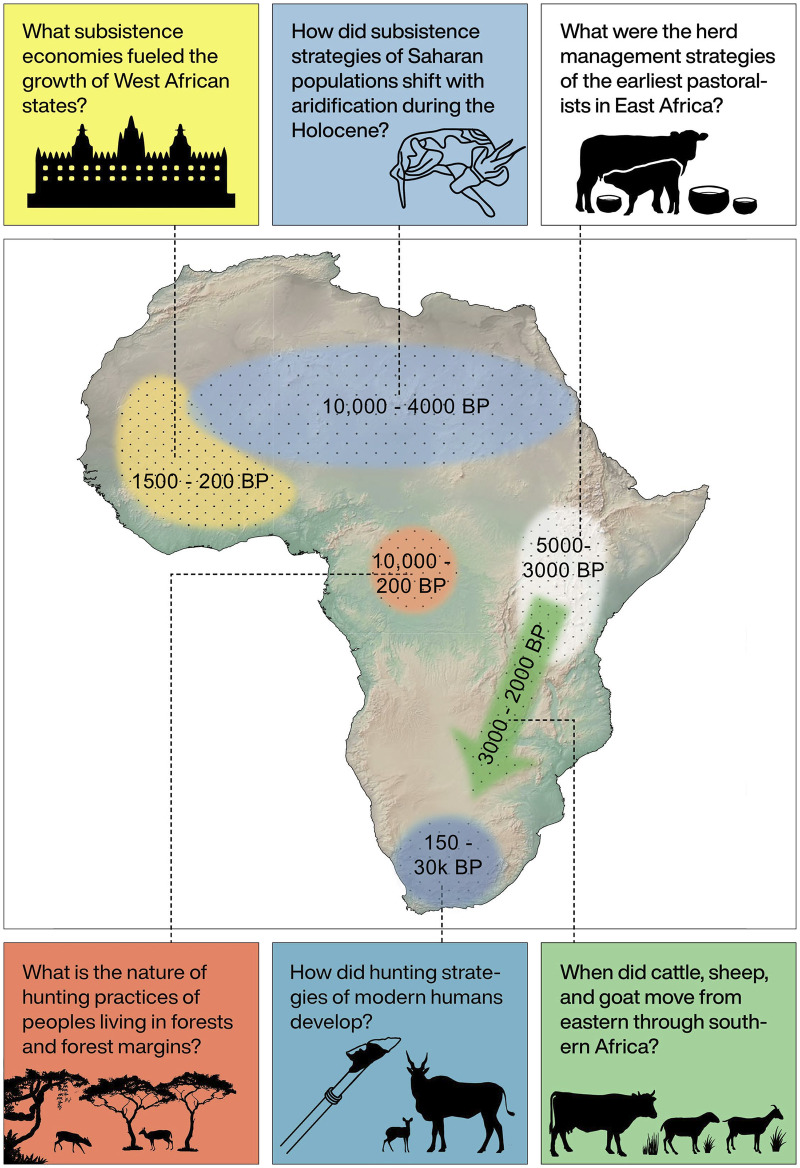
Examples of research questions in African archaeology that can be aided by ZooMS of bovids. Relevant time periods for each question are indicated. Elevation data from [19].

Assessment of prey choice and subsistence economies is not possible without secure taxonomic identifications. Bovid size classes have typically been used to assess broad hunting, transport, and carcass processing strategies [12, 15], but as bovids inhabit varying environments and exhibit behavioral differences, narrower identifications reveal more detail about forager decisions and tactics [23, 58, 62, 70–73] (Fig 4). Finally, bovid and mammal size classes used by zooarchaeologists across Africa vary [1, 58, 74], and therefore narrower identifications can aid in inter-regional comparisons of zooarchaeological data.

A major focus in Holocene African archaeology involves the study of the development of pastoral lifeways. Pastoralism in Africa is unique in that it developed much earlier than farming, first appearing in northern Africa around 8,000 years ago, and gradually spreading throughout the continent over the next several millennia. Tracking the spread of pastoralism and subsequent mixed agro-pastoralists across Africa, as well as exploring the diversity of pastoral management strategies across the continent, hinges on secure identifications of domesticates. Cattle, sheep, and goat expanded beyond the range of their wild progenitors: while there is some debate whether cattle were domesticated in Africa [75–78], wild African cattle (*Bos primigenius*) were restricted to northern Africa. Sheep and goat were domesticated in southwest Asia [79–82], and are the only members of subfamily Caprinae in sub-Saharan Africa. Still, identifying domesticates in archaeofaunal assemblages comes with some challenges. Cattle are osteologically similar to the widespread African buffalo (*Syncerus caffer*) [83], and while many osteological guides exist for identifying caprines [84–87], fragmentary post-cranial remains are difficult to distinguish from like-sized wild bovids [49].

Detailed analyses of herd management strategies have been carried out across eastern [3, 11, 88], and southern Africa [89–92], but somewhat patchily—large parts of the continent have not seen such studies (e.g. [93] in western Africa). Domestic caprines are also infrequently identified to species [45], precluding deeper analyses of herd management strategies and explorations of biological connections between communities (eg. [94]). A great deal of work remains to be done in establishing a firm chronology for the spread of domesticates across much of Africa, and major questions still exist regarding the mechanisms behind these dispersals, as well as the nature of pastoral economies over the last few millennia (e.g., [95]). Additionally, the exact trajectories of the expansion of herding throughout Africa are still poorly understood, as are the mechanisms by which herding took hold in different parts of the continent, i.e., whether through demographic migration or local adoption [25, 96]. Furthermore, herding was not adopted wholesale as it spread across the continent: clear archaeological evidence for the long-term persistence of foragers exists [58], and many food-producing populations also continued to rely on terrestrial wild taxa, as seen in Pastoral Neolithic [11, 97], as well as Iron Age sites in eastern and southern Africa [98–101]. In West Africa, recent studies highlight the range of wild bovid species exploited by humans until relatively recently, reflecting wild species’ roles in elite foodways, as seen in 17^th^ and 18^th^ century Dahomean palace sites [37] and revealed through investigation of the trans-Saharan trade in 11^th^ to 14^th^ century Senegal [102]. Animal diversity in ritual contexts, such as at Hierakopolis in predynastic Egypt [103] and 2^nd^ millennium CE rain control sites such as Ratho Kroonkop in South Africa [104], also highlights the need for a comprehensive set of reference markers for African fauna.

## ZooMS in African archaeology

To date, ZooMS analyses of faunal remains in Africa have been limited to a few pioneering studies. The first applications of ZooMS in African archaeological contexts were of ivory artifacts. Coutu et al. [46] used ZooMS to identify the species used in artifacts from Early Iron Age sites (c. 1400 to 1100 BP) in KwaZulu-Natal, South Africa, finding that elephants were the source of all ivory analyzed. Two recent studies have focused on bone artifacts: Desmond and colleagues [48] analyzed bone tools from the Later Stone Age cave site of Taforalt in eastern Morocco, and Bradfield and colleagues [47] used ZooMS to identify bone arrowheads from Iron Age (c. 1700 to 400 BP) sites in southern Africa. Both studies provided partial sets of reference markers for 6 and 13 bovids respectively, which allowed for coarse identifications of species used to manufacture bone artifacts.

Pastoral strategies have been explored by Prendergast et al. [45], who utilized ZooMS-based identifications to support and improve morphological identifications of sheep and goat at the Pastoral Neolithic site of Luxmanda in northern Tanzania. Le Meillour and colleagues [105] employed a paleoproteomic approach to identify bone fragments from the Later Stone Age sites of Toteng 1 and 3 in southern Africa. The authors demonstrate the usefulness of the method to identify suid, equid, and bovid (including domestic sheep) specimens. Prendergast et al. [44] used ZooMS to track species translocations. Circumventing the challenged posed by the osteological similarity between rat-sized native African murid species and introduced Asian black rats, ZooMS enabled significant improvement of species identifications. This study thus chronicled the late appearance of black rat in island and coastal East African sites in the second millennium BP, supporting the hypothesis that Asian species were introduced through Indian Ocean trade, but challenging ideas of early maritime-mediated biological exchange between Asia and Africa.

Most recently Coutu et al. [33] used ZooMS to separate domestic sheep from several similarly sized, wild bovids to confirm identifications of earliest domestic sheep in southern Africa. While only a small subset of bovids were analyzed, the development of a new marker that allows for separation between domestic and some wild bovids is critical in the use of ZooMS in Africa.

These initial studies highlight the potential of ZooMS in Africa, where, as in other parts of the world [42, 106–110], it has proven valuable in addressing a variety of questions. By providing identifications of morphologically ambiguous and fragmentary specimens, ZooMS has the potential to revive research interest in, and add value to, assemblages previously considered too poorly preserved for analysis. Through its role in clarifying taxonomic identifications, ZooMS opens new opportunities for addressing questions of paleoenvironment, subsistence strategies, foodways, the spread of and development of herding economies, and the symbolic importance of animals in the African past. However, application of ZooMS analysis across the continent has been hindered by a lack of complete reference peptide markers for African taxa, particularly bovids. Here we present the first comprehensive set of peptide markers for wild African bovids, based upon genetic and proteomic data from 34 different species across all subfamilies present in Africa. We also identify two additional markers that allow for further discrimination within subfamilies. Our results show that ZooMS can be used to distinguish among most bovid tribes, and in some cases provide narrower taxonomic identifications, which also allows for unambiguous identification of domestic livestock. We use these reference sets to identify wild and domestic taxa from six archaeological sites with extremely poor faunal preservation. These sites are located in southern and central Zambia, and span the Iron Age period of Zambia (c. 2000–500 years BP), an archaeological phase associated with the spread of farming, longer-term village settlements, and the formation of complex polities in southern Africa.

## Materials & methods

For proteomic analysis, modern reference samples were taken from twenty taxa across all six of the eight subfamilies present in Africa. In addition, to ensure that results were comparable with published markers, samples from modern cattle (*Bos taurus*) were also obtained to ensure similarity with previously published reference data for *Bos* (Table 1). Three individuals from each species were sampled from the Osteology Section at the National Museums of Kenya in Nairobi (all specimen information can be found in S1 Table). In the few cases where three individuals were not available for a species, at least three individuals were taken from the genus. 50 mg of bone from each individual was sampled using a handheld Dremel outfitted with a diamond drill bit at the Osteology Laboratory at the National Museums of Kenya, Nairobi. Between samples, the drill bits were sonicated and rinsed with distilled water. Published reference LC-MS/MS data for impala (*Aepyceros melampus*) and springbok (*Antidorcas marsupialis*) [33, 49] were used to confirm the presence of the correct marker sequences in these species, although MALDI-TOF-MS analysis was not conducted for these taxa in this study. Genetic data was mined from published Bovidae genomes (Chen et al, 2019, S1 File) for 28 African species. Combining both genomic and proteomic reference material, a total of 34 African species were analyzed. Genetic data from eight additional Bovid species and three species from two outgroups (Equidae and Cervidae) were also used along with previously published ZooMS markers (Table 1).

**Table 1:** List of the modern reference species used in this study.

Family	Subfamily	Tribe	Subtribe	Genus	Species	Common Name	DNA	ZooMS
Bovidae	Aepycerotinae			*Aepyceros*	*melampus*	impala	X^a^	*^d^^,^ ^g^
	Alcelaphinae			*Connochaetes*	*taurinus*	blue wildebeest	X^a^	X**^e^
				*Damaliscus (Beatragus)*	*hunteri*	hirola		X
				*Damaliscus*	*lunatus*	topi, tsessebe	X^a^	**X**
				*Alcelaphus*	*buselaphus*	hartebeest	X^a^	X**^e^^,^^f^
	Antilopinae	Antilopini		*Nanger*	*granti*	Grant’s gazelle	X^a^	**X**
				*Eudorcas*	*thomsonii*	Thomson’s gazelle	X^a^	**X**
				*Gazella*	*rufifrons*	red-fronted gazelle		**^d^^,^^f^
				*Antidorcas*	*marsupialis*	springbok	X^a^	X^g^^,^^h^
				*Litocranius*	*walleri*	gerenuk	X^a^	
		Neotragini		*Procapra*	*przewalskii*	Przewalskyi’s gazelle	X^a^	
				*Ourebia*	*ourebi*	oribi	X^a^	X**^e^
				*Oreotragus*	*oreotragus*	klipspringer	X^a^	X**^e^
				*Madoqua*	*kirkii*	Kirk’s dik-dik	X^a^	**X**
				*Neotragus*	*moschatus*	suni	X^a^	**X**
				*Raphicerus*	*campestris*	steenbok	X^a^	
	Pantholopinae			*Pantholops*	*hodgsonii*	Tibetan antelope	X^a^	*^d^
	Bovinae	Bovini	Bovina	*Bos*	*taurus*	cattle	X	X*^b^
				*Bos*	*grunniens*	yak	X^a^	
				*Bison*	*bison*	bison	X	*^d^
			Bubalina	*Bubalus*	*bubalis*	water buffalo	X	*^b^
				*Syncerus*	*caffer*	African buffalo	X^a^	**X**
		Tragelaphini		*Taurotragus*	*oryx*	common eland	X^a^	X**^e^
				*Taurotragus*	*derbianus*	giant eland		X
				*Tragelaphus*	*eurycerus*	bongo	X^a^	**X**
				*Tragelaphus*	*scriptus*	bushbuck	X^a^	
				*Tragelaphus*	*imberbis*	lesser kudu	X^a^	
				*Tragelaphus*	*strepsiceros*	greater kudu	X^a^	**^e^
				*Tragelaphus*	*buxtoni*	mountain nyala	X^a^	
				*Tragelaphus*	*spekii*	sitatunga	X^a^	
				*Tragelaphus*	*angasii*	nalya		**^e^
	Caprinae			*Ovis*	*aries*	sheep	X	*^b^^,^^c^^,^^d^^,^^h^
				*Capra*	*hirca*	goat	X	*^b^^,^^c^^,^^d^
				*Capra*	*ibex*	ibex	X^a^	*^b^^,^^c^
				*Ammotragus*	*lervia*	Barbary sheep		*^f^
	Cephalophinae			*Cephalophus*	*harveyi*	Harvey’s duiker	X^a^	
				*Philantomba*	*maxwellii*	Maxwell’s duiker	X^a^	
				*Sylvicapra*	*grimmia*	bush duiker	X^a^	**X**^h^
				*Cephalophus*	*adersi*	Aders’s duiker		**X**
	Hippotraginae			*Hippotragus*	*niger*	sable antelope		X**^e^
				*Hippotragus*	*equinus*	roan antelope		X**^e^^,^^f^
				*Oryx*	*gazella*	gemsbok	X^a^	
	Reduncinae			*Kobus*	*kob*	kob		**X**
				*Kobus*	*ellipsiprymnus*	waterbuck	X^a^	**X**
				*Redunca*	*redunca*	bohor reedbuck	X^a^	
Cervidae	Capreolinae			*Odocoileus*	*virginianus*	white tailed deer	X	*^d^
				*Rangifer*	*tarandus*	reindeer		*^d^
Equidae				*Equus*	*caballus*	horse	X	*^b^^,^^d^

ZooMS data was generated for three individuals from each of the species indicated, with LC-MS/MS conducted on one individual from the species indicated in bold. Members from Families Cervidae and Equidae with existing markers and sequence data were used to generate comparative reference spectra for validation. An ‘X’ in DNA column indicates species where genetic sequence data for COL1A1 and COL1A2 was available. An ‘X’ in the ZooMS column indicates species where data was generated for this publication. Bold indicates species analyzed by LC-MS/MS.

*Indicates where all 9 common ZooMS markers are published.

**Indicates where some, but not all of the common ZooMS markers are published.

^a^Genetic data from Chen et al (2019). Other genetic data was mined from NCBI.

^b^[38].

^c^[111].

^d^[112].

^e^[47].

^f^[48].

^g^LC-MS/MS data was analyzed from LeMeillour et al [49].

^h^ LC-MS/MS data was analyzed from Coutu et al. [33].

**Fig 5:**
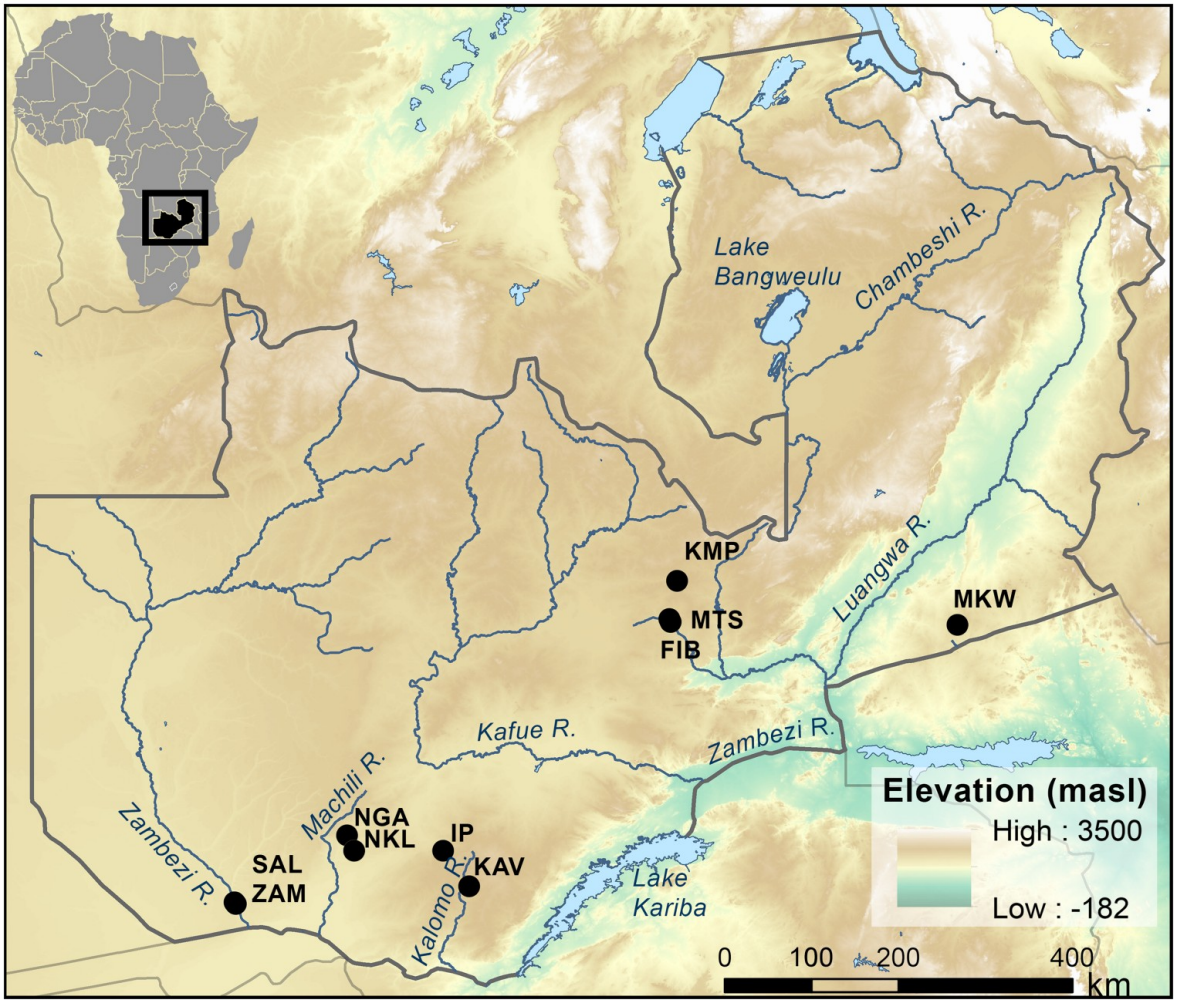
Location of archaeological sites in study. Salumano, SAL; Jakobo West, ZAM; Muteteshi, MTS; FIB, Fibobe II; Kapiri Mposhi, KMP; Kalundu Mound, KAV. Other sites mentioned in text include Isamu Pati, IP; Namakala, NKL; Nanga, NGA; and Makwe, MKW. Elevation basemap from [19].

Faunal specimens from the six archaeological sites included in this study are: Kapiri Mposhi A (n = 69), Fibobe II (n = 1), and Muteteshi (n = 4) in the Irumide Belt of Central Zambia, the Kalomo culture site of Kalundu Mound on the Batoka Plateau of southern Zambia (n = 102), and Salumano (n = 9) and Jakobo West (n = 7) on the margin of the Kalahari Depression of southwestern Zambia (Fig 5). All necessary permits were obtained for the described study for both modern and archaeological specimens, which complied with all relevant regulations. All sampled reference material is housed in the Osteology Section at the National Museums of Kenya in Nairobi (all specimen information can be found in S1 Table). Research and export permits in Kenya were granted by the National Commission for Science, Technology and Innovation (ref. NACOSTI/P/17/66411/14936), and the National Museums of Kenya (ref. NMK/ESC/ACL/j/VOL.1). Research and export permits for the samples were granted by NHCC, Lusaka, and fauna from Zambia sites were exported with permission of The Livingstone Museum. Faunal remains from these sites are highly fragmented and often poorly preserved due to culinary processing and unfavorable taphonomic conditions, particularly in the Kalahari sands at Salumano and Jakobo West [113].

Salumano is one of the type sites of the “Situmpa Group” of southwestern Zambia (also including Lusu Rapids, Situmpa, and Machili Forest Station), defined by Clark and Fagan [114] and Katanekwa [115] as reflecting a pre-Iron Age appearance of ceramics and livestock in the region. The early occurrences of Situmpa pottery and cattle at Salumano date to c. 2000 BP, overlaid by Early and Later Iron Age horizons extending into the last few hundred years [101, 116]. Salumano was revisited and re-tested by researchers at the Max Planck Institute for the Science of Human History and the Livingstone Museum in 2017, confirming this sequence. During the course of this research, a nearby locality of Jakobo West was discovered, which had only a single archaeological horizon with stone tools, bone fragments, and pot sherds that match typical “Situmpa Group” styles [115] encountered at about 1.3 m below surface.

Muteteshi and Fibobe II in the Mulungushi River Basin were first excavated by the Zambian National Monuments Commission between 1977 and 1979 under the direction of John Robertson [117]. Muteteshi is a single component Early Iron Age site with radiocarbon dates ranging from roughly 2000 to 1600 years BP, with early evidence of domesticated sorghum (*Sorghum bicolor*) [117]. Fibobe II is a nearby village site with substantial evidence for hut structures, iron working areas, and cultivation of domesticated crops dating to the Later Iron Age (approximately 1175–1410 years BP) [117]. Both sites were revisited in 2018 and the limited fauna analyzed here were recovered. Both sites had very poor faunal preservation.

Kapiri Mposhi A was identified during the course of new fieldwork in 2018. The site is not yet dated, but a transition from Later Stone Age to Iron Age material culture within a 4 meter sequence is apparent. Based on stone tool and ceramics styles, the site may have accumulated from c. 4000 to 600 years ago, likely by stone tool-using foragers who were in contact with Iron Age farmers [118].

Kalundu Mound is a roughly 140 x 106m low-sloped mound consisting of 3 meters of stratified archaeological material on the Batoka Plateau, southern Zambia. Excavations by Inskeep [119] and Fagan [98] identified Kalundu Mound as being diagnostic of the Later Iron Age ‘Kalomo Culture’ of the region based on ceramic styles, iron and ornamental artifacts, and burial traditions. Kalomo economies appear to have relied on sorghum cultivation and cattle-keeping, as well hunting of wild fauna [98]. However, current models for animal subsistence are based only on identification of teeth alone, and do not necessarily provide an accurate reflection of the breadth of animal species exploited during the Later Iron Age. Early radiocarbon dates on bulk charcoal samples from Kalundu Mound suggested use and formation of the mound occurred from ~1650–960 years ago, overlaying pit features that yielded dates in the mid 3^rd^ millennium BP. Re-excavation of a 1x3m trench and subsequent direct dating of macrobotanical and bone remains recovered in 2017 indicates a more precise date range of ~740–550 cal. BP based on Bayesian modeling of AMS radiocarbon dates on short-lived macrobotanical remains and bone fragments [120]. These excavations also yielded the largest faunal assemblage of the sites included in this study.

Before ZooMS analysis, each assemblage underwent a complete zooarchaeological analysis by AJ. This included all specimens that were either identifiable to element or measuring at least 20 mm. Each specimen was identified to element and portion, and to the narrowest taxonomic category (e.g. “Mammal”, “Bovid”, “*Bos* sp.”) based upon morphology. When possible, size was recorded, using Brain’s [1] bovid size classes. In most cases this was not possible, and thus the specimen was assigned to a minimum size class based upon thickness of cortical bone and identifiability relative to specimen size (e.g. mammal ≥2, indicating the specimen came from size 2 or larger mammal).

**Fig 6:**
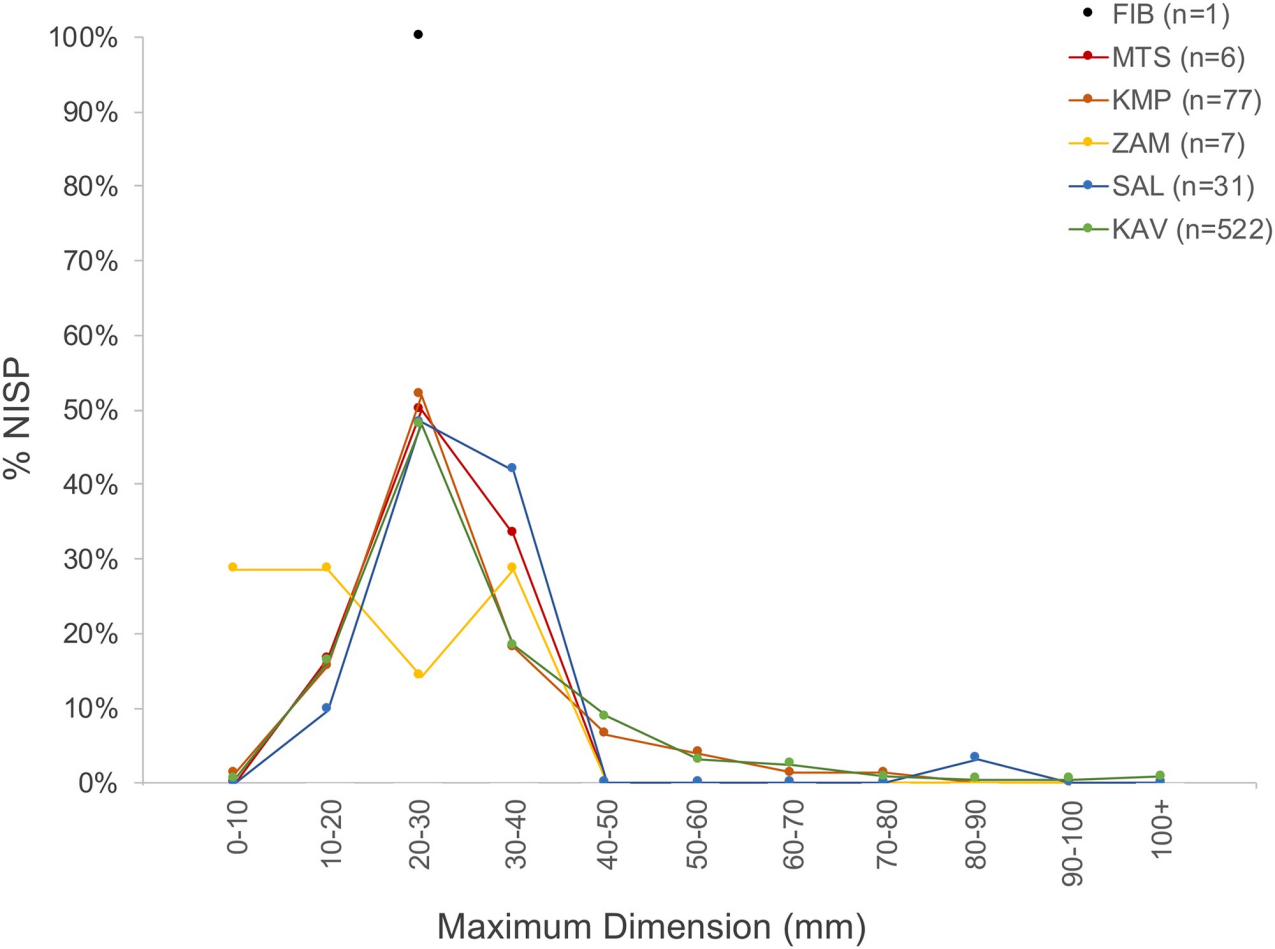
% NISP of maximum dimension of all identifiable fragments in 10cm bins. FIB, Fibobe II; MTS, Muteteshi; KMP, Kapiri Mposhi, SAL, Salumano; ZAM, Jakobo West.

The sites analyzed in this study have small faunal assemblages, ranging from 1 to 522 in number of identifiable specimens (NISP). Fragmentation rates are considerable at all sites (Fig 6)—the modal fragment size for all sites except for Jakobo West (ZAM, NISP = 7) is between 20 and 30mm. Such high rates of bone comminution rendered most fragments identifiable only to broader taxonomic groupings (S2 Table). ZooMS analysis of bone fragments was carried out on 100% of specimens from Jakobo West, 4 of 6 NISP from Muteteshi, 9 of 24 NISP from Salumano and 69 of 84 NISP from Kapiri Mposhi, excluding fragments that were clearly not mammalian, exhibited signs of burning, or were in such poor condition that collagen extraction was highly unlikely. Finally, 102 specimens, ~20% of the large assemblage from Kalundu (NISP = 522), were analyzed.

### Collagen extraction and digestion

For both archeological and modern bones, 10–20 mg of bone was subsampled and collagen was extracted based upon published ZooMS extraction protocols [38, 42, 121]. Bone powder or chips were demineralized in 500 μl of 0.5M hydrochloric acid at 4°C for one to two days. The supernatant was removed and the samples were rinsed twice with 50mM ammonium bicarbonate (NH_4_HCO_3_), pH 8.0 (AmBic). Then the samples were incubated in 100 μl of AmBic at 65°C for 1 hour. 50 μL of the resulting supernatant was incubated with 0.2 μg of trypsin (ThermoFisher Pierce™ Trypsin Protease) at 37°C overnight. Following digestion, the samples were acidified to 0.1% trifuoroacitic acid (TFA) and purified using a 100 μl C18 resin ZipTip^®^ pipette tip (EMD Millipore) with conditioning and eluting solutions composed of 50% acetonitrile and 0.1% TFA and a lower hydrophobicity wash buffer of 0.1% TFA. Peptides were eluted in 50 μl.

### Peptide mass fingerprinting

Tryptic collagen peptides from both modern and archaeological samples were diluted 1:10 with the eluting solution, and 0.5μl of the resulting tryptic peptides were mixed with a 0.5 μl of α-cyano-4-hydroxycinnamic acid matrix solution and spotted in triplicate along with calibration standards onto a ground steel 385 spot MALDI target plate. Samples were run on a Bruker Autoflex Speed MALDI-TOF mass spectrometer (Bruker Daltonics). Spectra were visually inspected using the mMass software [122] and low-quality spectra were rerun on the MALDI-TOF-MS without dilution or at 1:100 dilution depending upon the quality of the sample. MALDI-TOF-MS spectra for the modern references and protein sequence data can be found at Zenodo, doi:10.5281/zenodo.3964709 and for the archaeological samples at Zenodo, doi:10.5281/zenodo.3971142.

### LC-MS/MS

In order to confirm the sequences of peptide markers, one sample from each of the modern reference species was characterized by LC-MS/MS. The individuals chosen had the highest quality MALDI-TOF-MS spectra. 25μl of the tryptic peptide extract was sent for LC-MS/MS at the Functional Genomics Center Zurich using a Q-Exactive HF mass spectrometer (Thermo Scientific, Bremen, Germany) coupled with an ACQUITY UPLC M-Class system (Waters AG, Baden-Dättwil, Switzerland). For liquid chromatography, 0.1% formic acid in H2O was used for channel A and 0.1% formic acid in acetonitrile for channel B. Column temperature was 50°C. For each peptide sample, 4 μL was loaded onto the column with a flow rate of 15 μl/min of 99% solvent A for 30 seconds at room temperature. Eluting peptides were separated on a C18 column (HSS T3 C18, 100Å, 1.8 μm, 75 μm x 250 mm, Waters AG, Baden-Dättwil, Switzerland). After gradient stabilization for 1.5 min at 99% solvent A, peptides were separated over 120 minutes with a gradient of 1% - 5% solvent B in 30 seconds, 5% - 40% solvent B in 120 min. The column was cleaned after the run with 98% solvent B for 5 min and holding 98% B for 8 min prior to re-establishing loading condition. Full scan MS spectra (350–1500 m/z) were acquired in the Orbitrap with a resolution of 120’000 at 200 m/z, an automatic gain control target of 3e6, and maximum injection time of 50 ms. Precursors for MS2 scans were isolated with an isolation window of 1.2 Da, automated gain control of 1e5, a maximum fill time of 50 ms and HCD fragmented with a normalized collision energy of 28. From each MS1 scan, the 12 most intense precursor ions were fragmented and scanned with a resolution of 30’000 at 200 m/z and a fixed first mass of 130 m/z. For MS2 selection, the filter intensity threshold was 9e4. Unassigned singly charged ions and ions were excluded. Precursor masses already selected for MS/MS were excluded for further selection for 30 seconds. LC-MS/MS data is available at PXD020810 through MassIVE (doi:10.25345/C5TJ3M).

LC-MS/MS data was then quality checked using Byonic v.3.2.0 (Protein Metrics Inc. [123]) against SwissProt (downloaded 30 April 2019) with the following parameters: cleavage sites fully specific C-term R and K; 2 missed cleavages allowed; mass changes– 3 common, 1 rare; common: oxidation on M, P, and K, deamidation on N and Q; rare: pyro-Glu on N-term Q and E; no sequence variations allowed, wildcard search disabled, with decoys to confirm that the peptides were primarily Bovine collagen and to identify other significant protein hits. Proteins were considered present if they had 4 or more peptides with PEP 2D scores < 0.001 and a log probability > 5 (S3 and S4 Tables).

### Biomarker identification and confirmation

*Bos taurus* collagen protein sequences (P02453, P02465) were used to locate the collagen genes in the genomic data from bovids [124]. Collagen sequences were obtained from genomic data as nucleotide sequences, the exons were identified where possible and translated. Collagen sequences for all of the species were aligned in Geneious Prime 2019.0.4 (https://www.geneious.com). The locations of the nine well-characterized mammal markers [38, 42] were mapped onto the aligned sequences to predict the masses of these peptides in each species for which genetic data was available (S1 File). MALDI data for the reference samples, was peakpicked in mMass [122], with a signal to noise ratio of 6 and screened for peaks at the locations of the predicted masses. Markers were considered present for a species if they were found in 2 or more individuals at the predicted masses.

For confirmation of biomarkers, the MS/MS spectral data was then analyzed using Byonic with the following parameters: cleavage sites fully specific C-term R and K; 2 missed cleavages allowed; mass changes– 5 common, 1 rare; common: oxidation on M, P, and K, deamidation on N and Q; rare: pyro-Glu on N-term Q and E; no sequence variations allowed, wildcard search disabled, with decoys. The database was formed from the published sequences from the bovids (*Bos taurus* (P02453, P02465), *Bison bison* (XP_010841089.1, XP_010838069.1), *Bubalus bubalus* (XP_006041214.2, XP_006054012.1), *Ovis aries* (XP_011983013.1, XP_004007775.1), *Capra hircus* (XP_017920382.1, XP_005678993.1)), all of the mined bovid sequences from genetic data, the collagen sequences for outgroup species (*Odocolieus virginianus* (XP_020769668.1, XP_020769440.1), *Equus caballus* (XP_023508478.1, XP_001492989.1), the top proteins for the SwissProt search (168 proteins–S4 Table), and the common contaminants from Byonic. The peptide spectral matches (psms) to COL1 were filtered for an FDR < 1% and a PEP 2D score < 0.001. Markers were confirmed if there were at least three psms to the tryptic peptide corresponding to the genetic sequence of the marker with a precursor mass within 0.5 Da of the m/z identified in the MALDI spectra.

To identify new markers, the aligned bovine sequences were searched for sequence variants in Geneious. The masses of the tryptic peptides were predicted using PeptideMass from ExPASy [125, 126]. Peptides with masses between 800 and 3500 Daltons were then confirmed as authentic and the only peptide present at that mass in the LC-MS/MS results as above. MALDI-MS spectra were then analyzed for the consistent presence of any of these new candidate markers and only those which are present in over 75% of the reference spectra were presented as new diagnostic markers (S5 Table).

### Identification of archaeological samples

The archaeological spectra were first screened for good signal to noise and collagen signal by eye. Using mMass [122], high quality spectra were identified by presence of shared collagen markers. Spectra were processed using baseline correction (precision 52, relative offset 39), smoothing (Savitzky-Golay method with a window size of 0.3 m/z), deisotoping (maximum charge 1, isotope mass tolerance 0.1 m/z, isotope intensity tolerance 50%, isotope mass shift 0) and then peakpicked (signal to noise threshold of 3, relative intensity threshold 1%). The resulting peak lists were used to identify the samples to the lowest taxonomic level possible using the bovid markers.

## Results and discussion

### Data quality control

#### MALDI data

All of the modern reference samples yielded high quality MALDI data with the presence of shared collagen peaks and the 9 published markers. For the 192 archaeological samples analyzed, 62 were able to be identified, 44 yielded partial identifications, one yielded collagen was not identifiable to a known taxon, and 85 did not yield sufficient collagen for identification (S2 Table).

#### LC-MS/MS data

LC-MS/MS data from all of the species tested had over 85% coverage of both COL1 proteins S3 Table). 168 proteins were found during the analysis against SwissProt between all of the samples (S4 Table). These proteins were added to the col1 proteins for subsequent biomarker determination and confirmation. There was 100% agreement between the genetic data, the LC-MS/MS data, and the MALDI-TOF data for all of the markers presented here this includes the LC-MS/MS data from impala and springbok which aligns with the published genetic sequences for both species.

#### Common Bovidae ZooMS markers

The results of the reference specimens presented here broadly align with genetic studies of African bovids [124, 127–130]. All bovid species studied share four of the existing ZooMS markers: COL1α2 793–816 (D) at m/z 2131, COL1α2 454–483 (E) at m/z 2792, COL1α1 508–519 (P1) at m/z 1105, and COL1α2 292–309 (P2) at m/z 1648 (Table 2; S1 and S2 Figs). The other five existing ZooMS markers vary among bovid groups, and therefore combinations of these markers can be used to uniquely identify members of subtribe Bubalina, Bovina, tribe Tragelaphini, and subfamilies Hippotraginae, Alcelaphinae, Aepycerotinae, Reduncinae, and Cephalophinae. Several genera from the polyphyletic subfamily Antiliopinae can also be identified uniquely (Table 2; S1 and S2 Figs).

**Table 2:** ZooMS markers for Bovidae.

			COL1α1	COL1α2		COL1A2 375			COL1α2	COL1α2	COL1α2	COL1α2	COL1α2	COL1α2	COL1α1		COL1α2	
			508–519	978–990					484–498	889–906	502–519	292–309	793–816	454–483	586–618		757–789	
Subfamily	Tribe	Subtribe or Genus	P1	A	A’				B		C	P2	D	E	F	F’	G	G’
**Bovinae**	Bovini	Bovina**	1105	1192	1208	1182	2056	2072	1427	1532	1580	1648	2131	2792	2853	2869	3017	3033
**Bovinae**	Bovini	Bubalina	1105	1192	1208	1182	2056	2072	1455	1532	1580	1648	2131	2792	2853	2869	3059	3075
**Bovinae**	Tragelaphini	Group 1	1105	1192	1208	1182	2056	2072	1427	1560	1580	1648	2131	2792	2883	2899	3043	3059
**Bovinae**	Tragelaphini	Group 2*	1105	1192	1208	1182	2056	2072	1427	1532	1580	1648	2131	2792	2883	2899	3043	3059
Aepycerotinae**			1105	1180	1196	1182	2056	2072	1427	1532	1550	1648	2131	2792	2883	2899	3017	3033
**Antilopinae**	Antilopini	*Nanger*, *Eudorcas*, Antidorcas**	1105	1180	1196	1182	2056	2072	1427	1532	1550	1648	2131	2792	2883	2899	3017	3033
**Antilopinae**	Neotragini	Raphicerus*, *Ourebia*	1105	1180	1196	1182	2056	2072	1427	1532	1550	1648	2131	2792	2883	2899	3017	3033
**Antilopinae**	Neotragini	*Neotragus*	1105	1180	1196	1182	2056	2072	1427	1532	1580	1648	2131	2792	2883	2899	3017	3033
**Antilopinae**	Neotragini	*Oreotragus*	1105	1192	1208	1154	2028	2044	1427	1532	1580	1648	2131	2792	2883	2899	3017	3033
**Antilopinae**	Neotragini	*Madoqua*	1105	1166	1182	1182	2056	2072	1427	1532	1550	1648	2131	2792	2883	2899	3017	3033
**Cephalophinae**		*Sylvicapra*	1105	1192	1208	1182	2056	2072	1427	1532	1580	1648	2131	2792	2853	2869	3043	3059
**Cephalophinae**		*Cephalophus*	1105	1192	1208	1154	2028	2044	1427	1574	1580	1648	2131	2792	2853	2869	3043	3059
**Cephalophinae**		Philantomba*	1105	1192	1208	1154	2028	2044	1427	1560	1580	1648	2131	2792	2883	2899	3043	3059
**Reduncinae**			1105	1150	1166	1182	2056	2072	1427	1532	1550	1648	2131	2792	2883	2899	3017	3033
**Hippotraginae**			1105	1180	1196	1154	2028	2044	1427	1588	1580	1648	2131	2792	2883	2899	3043	3059
**Alcelaphinae**		*Alcelaphus*	1105	1180	1196	1154	2028	2044	1427	1590	1550	1648	2131	2792	2883	2899	3017	3033
**Alcelaphinae**		*Connochaetes*, *Damaliscus*	1105	1180	1196	1154	2028	2044	1427	1560	1550	1648	2131	2792	2883	2899	3017	3033
Pantholopinae*			1105	1180	1196	1182	2056	2072	1427	1560	1580	1648	2131	2792	2857	2873	3017	3033
**Caprinae**		Capra**	1105	1180	1196	1154	2028	2044	1427	1560	1580	1648	2131	2792	2883	2899	3077	3093
**Caprinae**		Ovis**	1105	1180	1196	1154	2028	2044	1427	1560	1580	1648	2131	2792	2883	2899	3017	3033

Masses in dark shaded cells need to be used carefully for interpretation. Masses in light shaded cells are present in the LC-MS/MS but are not suitable for species identification using MALDI. Both COL1α2 375–386 and COL1α2 375–396 are present in the LC-MS/MS and MALDI data, so both are presented. Group 1: *Taurotragus*, *Tragelaphus buxtoni*, *Tragelaphus euryceros*;

*Predicted from the genetic sequence data only, not confirmed with MALDI or LC-MS/MS data.

**Previously published species. *Neotragus pygmaeus* has markers different from the other *Neotragus* species predicted from the genetic data. See S1 File for more detail.

#### Novel ZooMS markers for bovids

Our results provide two new markers, COL1α2 375 and COL1α2 889–906 (see S1 File for discussion on marker nomenclature), that can be used to differentiate among several bovid groups. COL1α2 889–906 has six versions in the genetic data which was supported by LC-MS/MS analysis (Table 3). Two of these versions have identical masses and therefore cannot be distinguished using peptide mass fingerprinting techniques (S5 Table). The version found in most bovids has a peak at m/z 1532 [33]. Caprinae, Bovinae, some members of Alcelaphinae and Cephalophinae have peaks at m/z 1560. However, a peak at m/z 1560 cannot be used reliably for identification because another peptide at m/z 1560 is found in all bovids. COL1α2 889–906 is most useful for helping to distinguish Hippotraginae (m/z 1588). It also can be used to identify the genus *Cephalophus* (m/z 1574) within Cephalophinae, and to identify *Alcelaphus* (m/z 1590) within Alcelaphinae.

**Table 3:** Sequences of markers used to differentiate members of Bovidae.

Marker	Letter Name	Tryptic Name	Sequence	Mass	Mass’
COL1α2 978–990	A	A2T85	**A**GQPGAVGPAGIR	1150	1166
			**T**GQPGAVGPAGIR	1180	1196
			**I**GQPGAVGPAGIR	1192	1208
			**S**GQPGAVGPAGIR	1166	1182
COL1α2 375		A2T34	EGP**V**GLPGIDGR*	1182	
			EGP**V**GLPGIDGRPGPIGPAGAR*	2056	2072
			EGP**A**GLPGIDGR*	1154	
			EGP**A**GLPGIDGRPGPIGPAGAR*	2028	2044
COL1α2 484–498	B	A2T43	GIPGEFGLPGPAG**A**R	1427	
			GIPGEFGLPGPAG**V**R	1455	
COL1α2 889–906		A2T75	GEPGP**A**G**A**VGP**A**GAVGPR	1532	
			GEPGP**V**G**A**VGP**A**GAVGPR	1560	
			GEPGP**A**G**V**VGP**A**GAVGPR	1560	
			GEPGP**V**G**A**VGP**T**GAVGPR	1590	
			GEPGP**V**G**A**VGP**V**GAVGPR	1588	
			GEPGP**V**G**AI**GP**A**GAVGPR	1574	
COL1α2 502–519	C	A2T45	GPPGESGAAGP**A**GPIGSR	1550	
			GPPGESGAAGP**T**GPIGSR	1580	
COL1α1 586–618	F	A1T55/56	GLTGPIGPPGPAGA**P**GDKGE**A**GPSGPAGPTGAR	2853	2869
			GLTGPIGPPGPAGA**P**GDKGE**T**GPSGPAGPTGAR	2883	2899
			GLTGPIGPPGPAGA**A**GDKGE**T**GPSGPAGPTGAR	2857	2873
COL1α2 757–789	G	A2T67	GPSGEPGTAGPPGTPGPQG**L**LG**A**PGFLGLPGSR	3017	3033
			GPSGEPGTAGPPGTPGPQG**F**LG**P**PGFLGLPGSR	3077	3093
			GPSGEPGTAGPPGTPGPQG**L**LG**P**PGFLGLPGSR	3043	3059
			GPSGEPGTAGPPGTPGPQG**L**LG**L**PGFLGLPGSR	3059	3075

The sequences correspond to the masses for the diagnostic markers in Table 2. The names of the markers in different naming conventions used for ZooMS analysis in previous publications are also presented where relevant. Bold letters in the sequence data indicate locations of amino acid differences between the different versions of the markers. Masses in dark shaded cells need to be used carefully for interpretation. Masses in light shaded cells are present in the LC MS/MS, but are not suitable for species identification using MALDI.

*Both the fully cleaved peptide and the peptide with one missed cleavage are present in the LC-MS/MS and the MALDI.

Marker COL1α2 375 is more complicated to use as tryptic digestion is sometimes incomplete when the digestion site is followed by a proline (when indicating both markers only the first position is used in the name). This is illustrated by the presence of peaks corresponding to peptides with no missed cleavages (COL1α2 375–386) and one missed cleavage (COL1α2 375–396). Bovinae, Reduncinae, and some members of Antilopinae and Cephalophinae have peaks corresponding to COL1α2 375–396 (m/z 2056/2072) that cannot be used reliably for identifications because they are composed of several peptides. COL1α2 375–386 (m/z 1182) can also overlap with the COL1α2 978–990 (A’) marker present at the same m/z value in some species of Neotragini (e.g. *Madoqua*). Therefore, caution is needed when using m/z 1182. However, m/z 1154 (COL1α2 375–386) and m/z 2028/2044 (COL1α2 375–396) can be used to uniquely identify Caprinae, Alcelaphinae, and Hippotraginae. The presence of peaks at m/z 1154/2028/2044 can also be used to identify the genera *Cephalophus* and *Philantomba* within Cephalophinae and *Oreotragus* within Antilopinae.

#### G peptide (COL1α2 757–789)

**Fig 7:**
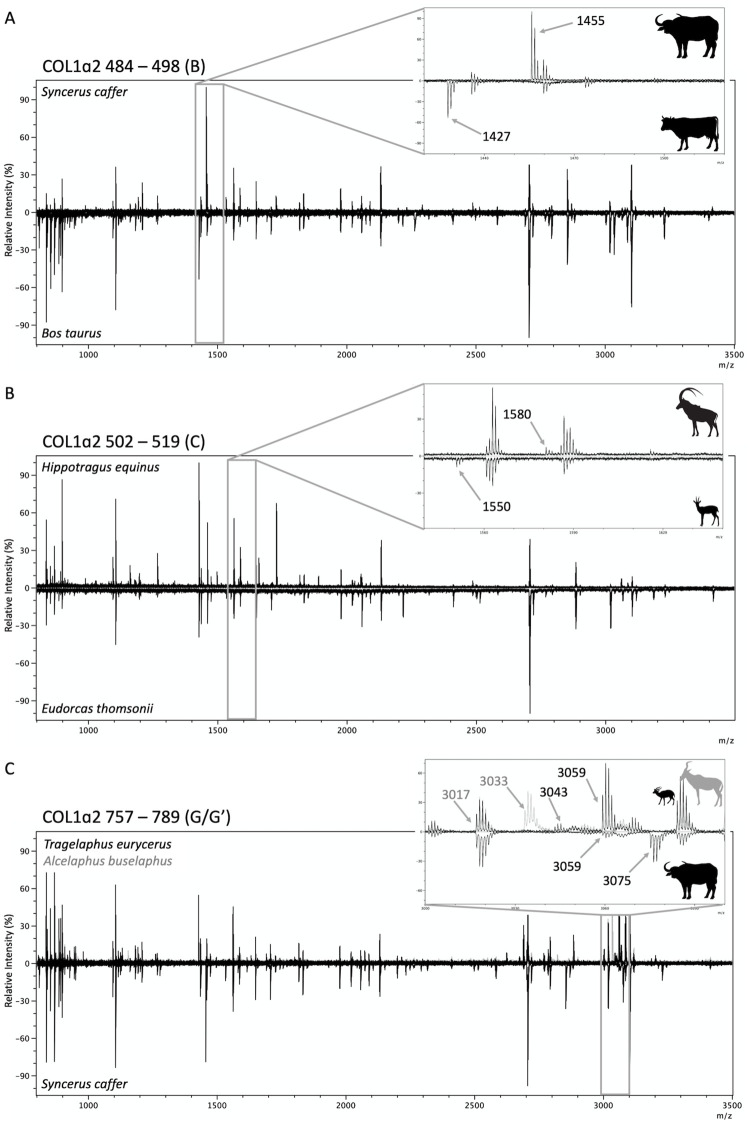
Examples of differences between bovid groups for some published marker sets. Panel A shows the difference between the COL1α2 484–498 (B) marker at m/z 1455 for *Bubalina* (shown: *Syncerus caffer*) and m/z 1427 for all other bovids (shown: *Bos taurus*). Panel B shows the difference between the COL1α2 502–519 (C) marker at m/z 1580 (shown: *Hippotragus niger*) and at m/z 1550 (shown: *Eudorcas thomsonii*). Panel C shows the difference between the COL1α2 757–789 (G/G’) marker at 3033 (shown: *Alcelaphus buselaphus*, G’ only), m/z 3043/3059 (shown: *Tragelaphus eurycerus*), and m/z 3059/3075 (shown: *Syncerus caffer*). The peak at m/z 3017 is composed of multiple peptides, one of which is shared between all Bovids (shown in *Syncerus caffer*), and therefore should not be used for identification.

The high mass COL1α2 757–789 (G and G’) peptides are commonly used for identification of sheep and goat [111]. They also show diversity among other bovid groups. The version seen in sheep and cattle has peaks at m/z 3017 and 3033. Frequently however, a peak at m/z 3017 (but not m/z 3033) has been observed in *Capra* spectra along with their unique markers (m/z 3077/3093) (Fig 7; S3 Fig). In investigating the cause for this, our LC-MS/MS data showed that another peptide, conserved across all of the bovid species as shown from the sequence data, is present at m/z 3017 (GPPGASGAPGPQGFQGPPGEPGEPGQTGPAGAR–COL1α2 89–130) (S3 Fig). This not the same peptide used in the identification of sheep/cattle and is not a reliable marker for ZooMS identification. Therefore, we suggest that m/z 3017 should no longer be used in the identification of bovids as there is a high likelihood for misidentification. The peak at m/z 3033, should still be considered a reliable marker for COL1α2 757–789 (G’) and can continue to be used for identification without its counterpart.

### Distinguishing among bovid groups

#### Subfamily Bovinae

All members of the subfamily Bovinae share the COL1α2 978–990 (A/A’) marker at m/z 1192/1208 and the COL1α2 502–519 (C) marker at m/z 1580. Within Bovinae are two tribes, Tragelaphini and Bovini, which can be distinguished from each other using the COL1α1 586–618 (F/F’) marker with m/z of 2853/2869 for Bovinae and m/z 2883/2869 for Tragelaphini, and the COL1α2 757–789 (G’) marker with an m/z of 3033 for Bovinae and 3043/3059 for Tragelaphini (Table 2). Both Tragelaphini and Bovini share the new marker COL1α2 375 (m/z 1182/2056/2072), but within Tragelaphini there is variation in COL1α2 889–906. *Tragelaphus imberis* (lesser kudu), *Tragelaphus scriptus* (harnessed bushbuck), *Tragelaphus spekii* (sitatunga), and *Tragelaphus strepsiceros* (greater kudu) share the variation at m/z 1530. *Tragelaphus buxtoni* (mountain nyala), *Tragelaphus euryceros* (bongo) and both species of *Taurotragus* (elands) have a peak at m/z 1560; so care should be taken when using this marker to distinguish between tragelaphines, as the latter is not a reliable indicator. In addition, because there is variation with the genus *Tragelaphu*s, only the specific species reported here can be identified using COL1α2 889–906.

Bovini includes two subtribes of which members have known peptide markers: Bovina, containing *Bos* and *Bison* and Bubalina, containing *Bubalus* (water buffalo and their relatives) and *Syncerus caffer* (African buffalo). We confirm that *Syncerus* shares the same COL1α2 484–498 (B) marker at m/z 1455 and COL1α2 757–789 (G/G’) markers at m/z 3059/3075 with *Bubalus* [38, 112], allowing for African buffalo to be distinguished from cattle.

#### Subfamilies Reduncinae and Hippotraginae

**Fig 8:**
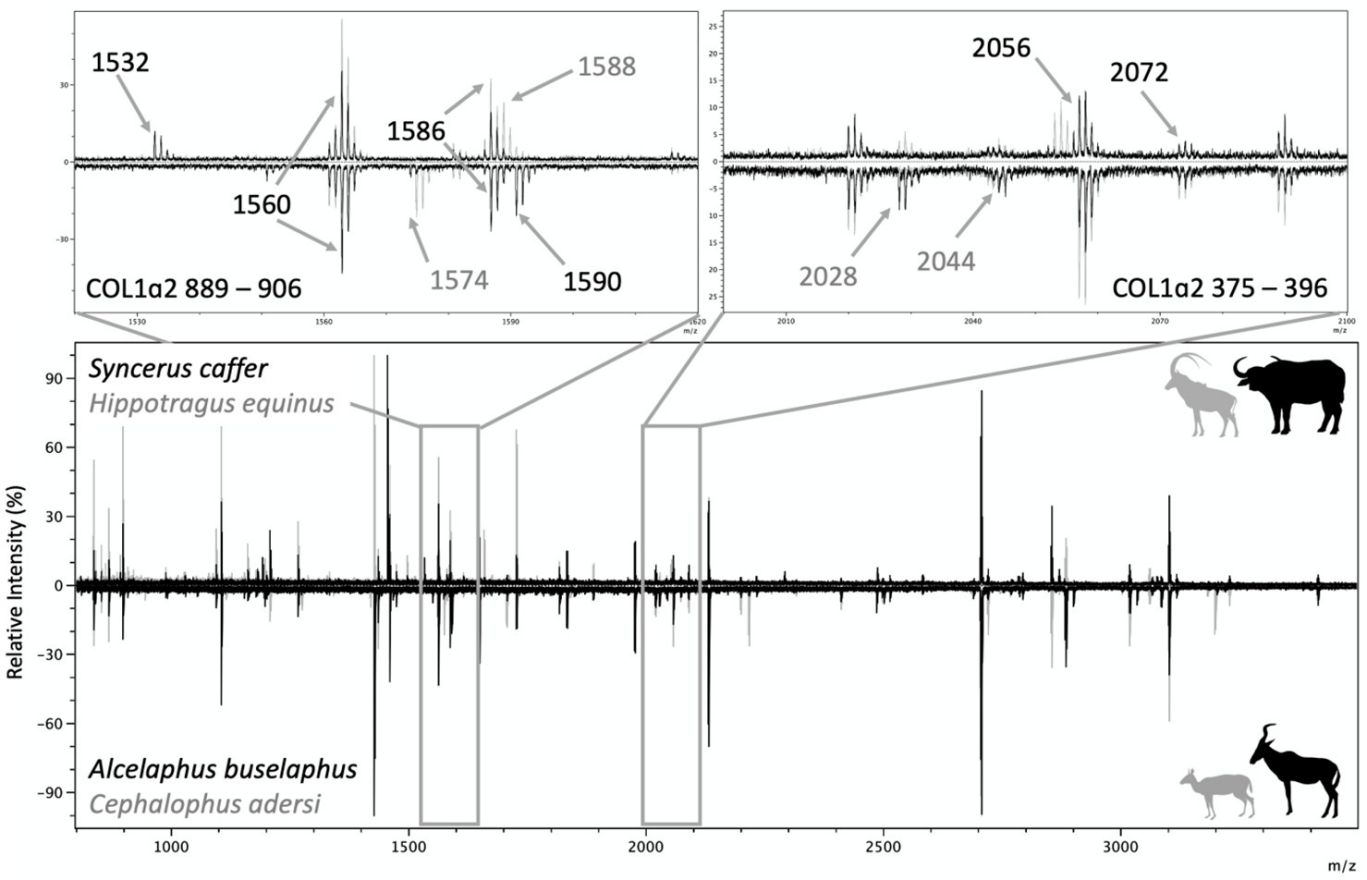
Novel markers shown for four different families. Most bovids share the COL1α2 889–906 marker at m/z 1532 (shown: *Syncerus caffer*). Hippotraginae can be distinguished using m/z 1588. However, this must be used carefully as the marker overlaps with the peak shared by all species at m/z 1586 (shown: *Hippotragus equinus*). Members of the genus *Cephalophus* can be identified based upon the COL1α2 889–906 marker at m/z 1574 (shown: *Cephalophus adersi*) and the genus *Alcelaphus* can be identified from other Alcelaphines at m/z 1590 (shown: *Alcelaphus buselaphus*). The COL1α2 375–396 marker is shown (m/z 2056 and 2072, shown in *Hippotragus equinus*). Subfamilies Hippotraginae, Caprinae, and Alcelaphinae, along with *Oreotragus* and some genera of Cephalophinae can be distinguished with markers at m/z 2028 and m/z 2044. *Syncerus caffer* has peaks at m/z 2056/2072, which are shared with another peptide present in all bovids and therefore not suitable for diagnostic purposes.

Members of Reduncinae can be distinguished from all other bovid groups by the COL1α2 978–990 (A/A’) markers at m/z 1150/1166. In Reduncinae the COL1α2 502–519 (C), COL1α1 586–618 (F/F’), and COL1α2 757–789 (G/G’) markers are shared with several other groups, with a peak at m/z 1550, m/z 2883/2899, and m/z 3043/3059, respectively (Table 2). Members of Hippotraginae share COL1α2 978–990 (A), COL1α2 484–498 (B), COL1α2 502–519 (C), COL1α1 586–618 (F), and COL1α2 757–789 (G) markers with other groups, but can be uniquely identified with the new marker COL1A2 889 at m/z 1572 and 1588 (Table 2). Caution should be used with m/z 1588 as it overlaps with a shared collagen peptide at m/z 1586 (Fig 8). In addition, the combination of the COL1α2 978–990 (A/A’) marker at m/z 1180/1196 and the COL1α2 757–789 (G/G’) marker at m/z 3043/3059 can also be used to uniquely identify Hippotraginae.

#### Subfamily Alcelaphinae

All Alcelaphinae analyzed in this study share COL1α2 978–990 (A/A’) marker at m/z 1180/1196, COL1α2 502–519 (C) marker at m/z 1550, COL1α1 586–618 (F/F’) at m/z 2883/2899, and COL1α2 757–789 (G/G’) at m/z 3017/3033, and thus they are indistinguishable from *Aepyceros melampus* (impala), *Nanger granti* (Grant’s gazelle), *Eudorcas thomsonii* (Thomson’s gazelle), and *Ourebia ourebi* (oribi) using only the existing markers. However, all alcelaphine bovids have COL1α2 375 at m/z 1154/2056/2072, which can be used to distinguish them uniquely. Furthermore, the new COL1α2 889–906 marker at m/z 1590 can be used to uniquely identify the genus *Alcelaphus* (hartebeest). The two other genera we analyzed, *Connochaetes* (wildebeest) and members of the genus *Damaliscus* (topi and tsessebe) have m/z 1560, which is not useful for identification.

#### Subfamily Cephalophinae

We only analyzed members of two genera, *Cephalophus* and *Sylvicapra*, which share all of the existing ZooMS markers with members of the subtribe Bovina except for the COL1α2 757–789 (G/G’) marker, which in Cephalophinae has an m/z of 3043/3059 (Table 2). *Cephalophus adersi* (Aders’s duiker) and *Sylvicapra grimmia* (bush duiker) share the same existing marker profile but can be distinguished based upon the two novel markers. *Cephalophus adersi* can be identified by COL1α2 375 at m/z 1182/2056/2072 and COL1α2 889–906 at m/z 1574 and *Sylvicapra grimmia* by COL1α2 889–906 at m/z 1532 (Table 2). Genetic data for a third species, *Philantomba maxwellii* (Maxwell’s duiker), indicates that this genus could be uniquely identified using the COL1α1 586–618 (F/F’) and COL1α2 375 markers. While we consider all three genera within the subfamily Cephalophinae here, many species have not been assessed, particularly within *Cephalophus* which includes 15 extant species. These taxa are also widespread across the continent, particularly in forest zones (Fig 1). Given the differences we have presented here and the diversity within Cephalophinae [131] it is possible that other members of Cephalophinae might have different marker profiles (e.g. different masses for various markers), so care should be taken when identifying new species of Cephalophinae, verifying ZooMS markers with LC-MS/MS and/or genetic data.

#### Subfamily Antilopinae

There is considerable diversity within Antilopinae. Traditionally members of this subfamily have been grouped into two tribes: Antilopini (gazelles and their relatives)—for which the specimens analyzed in this study include *Eudrocas thomsonii* (Thomson’s gazelle) and *Nanger granti* (Grant’s gazelle)—and Neotragini (African dwarf antelopes), for which the specimens analyzed in this study historically has included *Ourebia ourebi*, *Neotragus* spp., *Madoqua* (dik-diks), and *Oreotragus* (klipspringer). However, genetic studies have shown that Antilopinae is actually polyphyletic [124, 127, 132]. In particular, the tribe Neotragini has long been considered a catch-all group for small antelopes, which is reflected in the lack of consistent markers for the entire tribe with four different marker profiles for the five genera studied.

All Antilopinae share the same COL1α1 586–618 (F/F’) markers at m/z 2883/2899, COL1α2 757–789 (G’) marker at m/z 3033, and COL1α2 889–906 marker at m/z 1532. All studied Antiopini and some of the Neotraini (genera *Ourebia* and *Raphicerus*) all share the existing marker profiles with Alcelaphinae and *Aepyceros* (Table 2*)*. While COL1α2 375 allows Alcelaphinae to be uniquely identified, Antilopini, *Ourebia*, and *Raphicerus* have the marker at m/z 1182/2056/2072 and cannot be distinguished. Several of the neotragine bovids have unique profiles. *Oreotragus oreotragus* (klipspringer) is highly divergent from the other Neotragini with the COL1α2 978–990 (A/A’) marker at m/z 1192/1208, COL1α2 502–519 (C) marker at m/z 1580, and the COL1α2 375 marker at m/z 1154/2028/2044. *Madoqua kirkii* (Kirk’s dikdik) has a unique COL1α2 978–990 (A/A’) marker at m/z 1166/1182, distinguishing it from all other bovid groups (Table 2). *Neotragus moschatus* (suni) can be distinguished from the other Antilopinae species with a combination of COL1α2 978–990 (A/A’) markers at m/z 1180/1196 and COL1α2 502–519 (C) marker at m/z 1580. Although *Neotragus moschatus* and *Ovis* share identical existing marker sets, they can be uniquely distinguished by the presence of the COL1α2 375 marker in *Ovis* at m/z 1154/2028/2044 and the COL1α2 889–906 marker in *Neotragus moschatus* at m/z 1532. Given the diversity within the Antilopinae, the markers are indicated here only for the species tested. Further sampling for ZooMS and LC-MS/MS analysis will need to be done to confirm markers of other taxa within this subfamily, such as gerenuk (*Litocranius walleri*) and steenbok and grysbok (genus *Raphicerus*).

The results of this study show that it is possible to distinguish among major bovid groups and securely identify domestic livestock from wild bovids in sub-Saharan Africa using ZooMS [38]. In some cases, body size can also be used to refine ZooMS identifications when only partial ZooMS spectra are recovered (see also [47]). For example, *Bos*, *Cephalophus*, and *Sylvicapra* share all markers but the COL1α2 757–789 (G’) peptide. The substantial size difference between *Bos* and members of Cephalophinae can be used to differentiate these taxa in cases when the COL1α2 757–789 (G’) peptide is not preserved, which is a common occurrence (S2 Table).

In some cases identification is more difficult. *Aepyceros melampus*, *Nanger granti*, *Eudorcas thomsonii*, *Ourebia ourebi*, and *Raphicerus* all share the same suite of peptide markers. Impala and Grant’s gazelle are similar in size, and while both are larger than *Eudorcas*, *Ourebia*, and especially *Raphicerus* [52], it is likely that fragmentary bone—not identifiable to element—would not be attributable to a size class, and therefore will not be useful in refining ZooMS-based identifications. The presence of juvenile specimens presents another confounding factor, particularly in cases where bone fragments are not identifiable to element.

### Comparison with published markers

Our study provides a comprehensive set of markers for the bovid groups, and our results compare favorably with published markers in other studies. Our results agree with the incomplete published marker profiles for *Alcelaphus buselaphus* [48], members of Antilopini from [48, 49, 112], and *Antidorcas marsupialis* and *Sylvicapra grimmia* from [33]. Bradfield and colleagues [47] present incomplete marker profiles for a number of bovid species that were unconfirmed with LC-MS/MS. In three cases our markers differ from those presented in [47]. We confirm using both sequence and LC-MS/MS data that *Connochaetes taurinus* (blue wildebeest) has the same existing marker profiles as the other alcelaphines, contradicting the differences in the COL1α2 978–990 (A) and COL1α2 757–789 (G) markers reported by Bradfield et al. [47]. In our work all Hippotragine species tested have the same COL1α2 757–789 (G/G’) markers at m/z 3043/3059. In addition, we do not find the differences in the existing markers between Tragelaphini species as reported by Bradfield et al. [47]. We analyzed predicted protein sequences and/or LC-MS/MS data for two species from the genus *Taurotragus* and six from *Tragelaphus*, including two of the three species that were analyzed in [47] and find that the COL1α2 757–789 (G) makers are identical across all eight Tragelaphini species tested. The difference in the COL1α2 757–789 (G) maker profiles may be related to the difficulty of using the peak at m/z 3017 which is why we recommend that it no longer be used to distinguish between bovid species. Our LC-MS/MS analysis of the data from Le Meillour et al. [49] agrees with that of Coutu et al. [33] in that we have identified the same inconsistencies in the LC-MS/MS data and their published full sequence. We do not challenge species identifications proposed by Le Meillour et al. (2020), only additional parts of the sequence data which were not used for their identifications, but our used in this study and in Coutu et al. [33]. These differences highlight the importance of confirming markers using sequence and/or LC-MS/MS data to ensure accuracy.

### ZooMS analysis and archaeofaunal identifications

Collagen preservation among the sampled archaeological sites is extremely uneven. Eight of nine specimens analyzed from Salumano yielded good spectra and taxonomic identifications, but none of the four analyzed specimens from Muteteshi yielded any collagen, and only eight of 69 specimens analyzed from Kapiri Mposhi yielded complete spectra for identification beyond family, while another three were identifiable to other families (e.g. Leporidae) (Table 4). Forty-one of 102 specimens from Kalundu yielded good spectra sufficient for identifications (Table 4). Only two of the Kalundu specimens were not attributable to any known taxa on the basis of ZooMS. One of the four good spectra obtained from the seven analyzed Jakobo West specimens was not identifiable to a known taxon. Finally, the single specimen analyzed from Fibobe yielded identifiable results. The detailed results for all sites are presented in S2 Table (Table 4). In sum, c. 44% of analyzed specimens failed to yield collagen. A range of factors likely contributed to the lack of collagen preservation at some sites. Kalahari sands potentially impacted preservation at Jakobo West, consistent with other studies of collagen preservation in sandy sediments [113], although nearby Salumano yielded better results. More generally, collagen preservation in tropical areas can be poor due to high temperatures, fluctuations in temperature, and a dynamic hydrologic environment [133, 134]. Culinary processing, including boiling, may also be responsible for loss of collagen [135]. Future ZooMS analyses would benefit from additional screening techniques to assess collagen preservation prior to collagen extraction and analyses [105, 109, 136].

**Table 4:** Identifiable/analyzed versus unidentifiable bone recovered from each site by weight in grams, and number of specimens analyzed and number of successful, partial, and failed spectra.

Site	Bone recovered from each site by weight in grams			Results of ZooMS analysis (number of specimens)				
	ID/analyzed	NID	Total	Analyzed	Full ID	Full spectra (unknown animal)	Partial spectra and ID	Fail
**Kapiri Mposhi**	119.1	7.4	126.5	69	11	1	12	45
**Muteteshi**	5.1	0	5.1	4	–	–	–	4
**Fibobe**	1.1	0	1.1	1	–	–	1	–
**Salumano**	58.5	11.8	70.6	9	7	–	1	1
**Kalundu**	1280.2	185.7	1465.9	102	41	–	29	32
**Jakobo West**	6.9	1.0	7.9	7	3	1	–	3

For bone recovered from the site, “ID/analyzed” includes the fraction of the assemblage that measured at least 20 mm in maximum dimension or was either identifiable to element or taxon. “NID” includes all other faunal material that was unidentifiable and measuring under 20 mm. “Full ID” includes bovids identified to subfamily or narrower taxonomic grouping, as well as wild species identified to family (e.g. Mustelidae, Leporidae). “Full spectra (unknown animal)” includes specimens that yielded good spectra but could not be attributed to any taxon because many African taxa do not have published peptide markers. “Partial spectra and ID” includes specimens that yielded some peptide markers, but not enough for definitive identifications. “Fail” includes specimens that did not produce any peptide markers.

**Fig 9:**
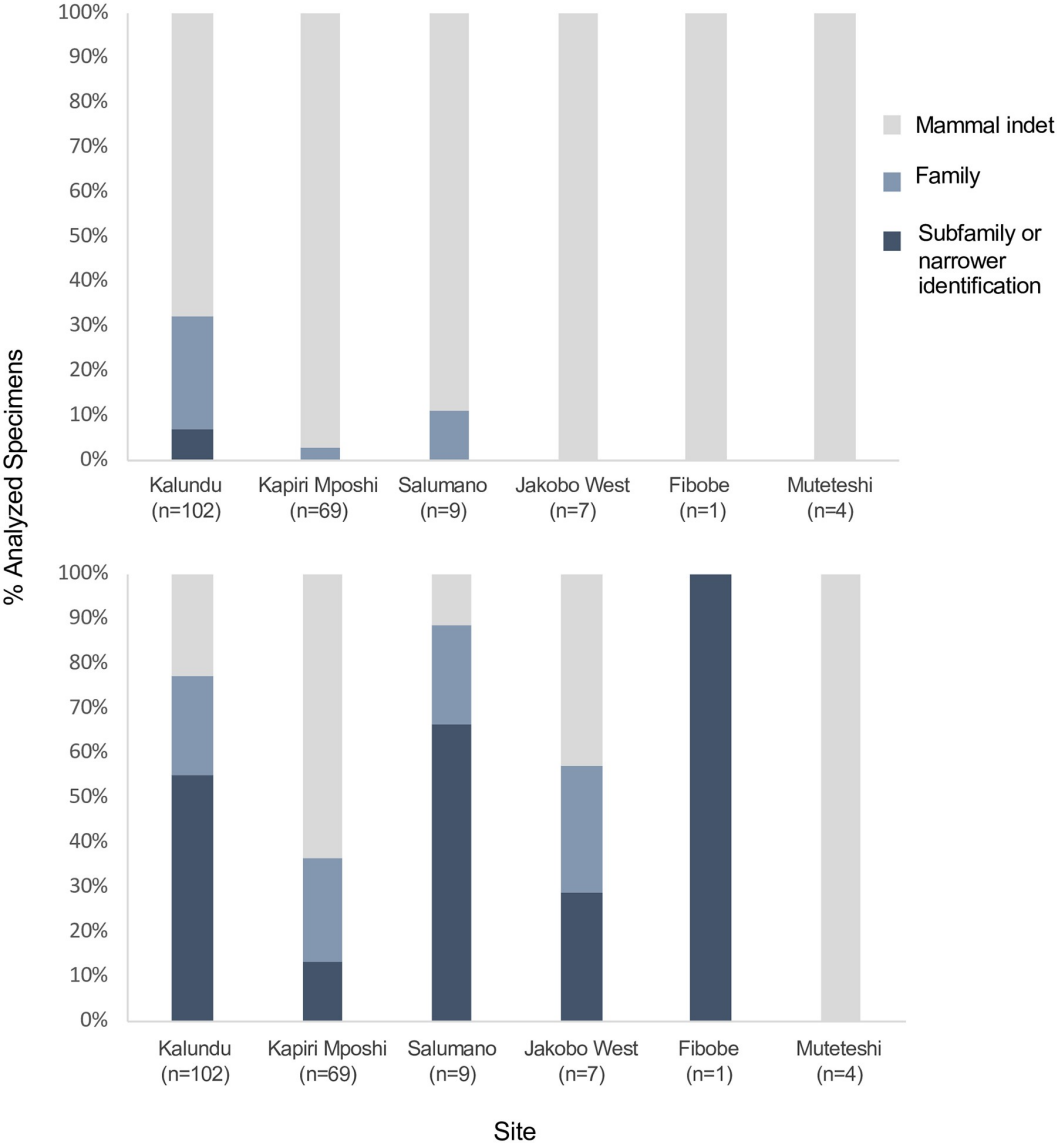
Percent of sampled specimens identified to broad taxonomic categories. Upper chart shows identifications based on morphology alone. Lower chart shows identifications using ZooMS and morphology.

Specimens yielding full and partial spectra spectra revealed considerably more taxonomic diversity at the sites than did morphological identifications. In all sites except for Muteteshi—in which all four specimens failed to yield sufficient or well-preserved collagen for ZooMS-based taxonomic identification—taxonomic richness (here NTAXA includes bovid group or other family such as Canidae or Leporidae) increased with ZooMS analysis (Fig 9; Table 5). Analysis of the archaeofaunal remains show that, in some cases, ZooMS analysis of even the smallest assemblages can reveal greater taxonomic richness than morphological identifications. Analysis of only 9 specimens from Salumano resulted in identification of three taxa. The two largest assemblages, Kalundu and Kapiri Mposhi, also saw increases in NTAXA. Better preservation at Kalundu resulted in the greatest NTAXA increase, from 3 to 10. Poor preservation at Kapiri Mposhi resulted in many failed samples and several partial identifications (e.g., Aepycerotinae/Alcelaphinae/*Nanger*/*Eudorcas*/*Raphicerus*/*Oribi*). However, because these specimens were originally identified as either indeterminate mammals or bovids, these partial identifications are still an improvement over identifications based solely upon morphology, and demonstrate the presence of wild bovids at the site (S2 Table).

**Table 5:** Identifications of analyzed archaeological specimens.

Taxon	KAV Morph	KAV ZooMS + Morph	KMP Morph	KMP ZooMS + Morph	SAL morph	SAL ZooMS + Morph	ZAM Morph	ZAM ZooMS + Morph	FIB Morph	FIB ZooMS + Morph	MTS Morph	MTS ZooMS + Morph
**Subfamily/tribe**	**7**	**56**	**0**	**9**	**0**	**6**	**0**	**2**	**0**	**1**	**0**	**0**
*Bos*	–	35	–	7	–	5	–	2	–	1	–	–
Cf. *Bos*	5	5	–	–	–	–	–	–	–	–	–	–
Tragelaphini	–	2	–	–	–	1	–	–	–	–	–	–
Cephalophinae cf. *Sylvicapra*	–	4	–	–	–	–	–	–	–	–	–	–
Cephalophinae	–	5	–	–	–	–	–	–	–	–	–	–
Hippotraginae	–	2	–	–	–	–	–	–	–	–	–	–
Reduncinae	–	–	–	2	–	–	–	–	–	–	–	–
Antilopini: *Eudorcas/Ourebia*	–	–	–	–	–	–	–	–	–	–	–	–
Caprinae	1	1	–	–	–	–	–	–	–	–	–	–
Elephant	–	1	–	–	–	–	–	–	–	–	–	–
Rhinoceros	–	1	–	–	–	–	–	–	–	–	–	–
Cf. Human	1	–	–	–	–	–	–	–	–	–	–	–
**Family**	**26**	**23**	**4**	**17**	**1**	**2**	**0**	**2**	**0**	**0**	**0**	**0**
Mustelidae	–	–	–	1	–	–	–	–	–	–	–	–
Canidae	–	2	–	–	–	–	–	–	–	–	–	–
cf. Suid	–	1	–	–	–	1	–	–	–	–	–	–
Bos/Tragelaphini size ≥3	–	2	–	–	–	–	–	–	–	–	–	–
Bos/Cephalophinae	–	1	–	1	–	–	–	–	–	–	–	–
Bos/Cephalophinae cf. *Sylvicapra*	–	4	–	3	–	–	–	2	–	–	–	–
Bos/Tragelaphini/Cephalophinae	–	–	–	1	–	–	–	–	–	–	–	–
Bos/Tragelaphini/Cephalophinae cf. *Sylvicapra*	–	–	–	–	–	–	–	–	–	–	–	–
Hippotraginae/Tragelaphini	–	1	–	–	–	–	–	–	–	–	–	–
Eudorcas/Ourebia	–	1	–	–	–	–	–	–	–	–	–	–
*Bos*/Tragelaphinae/*Oreotragus*/Cephalophinae	–	1	–	–	–	–	–	–	–	–	–	–
Aepycerotinae/Alcelaphinae/Antilopinae/Caprinae/ Hippotraginae	–	–	–	1	–	–	–	–	–	–	–	–
Aepycerotinae/Antilopinae/Reduncinae/Caprinae—*Ovis*	–	1	–	–	–	–	–	–	–	–	–	–
Alcelaphinae/Aepycereotinae/*Nanger/Eudorcas/Ourebia*	–	–	–	1	–	1	–	–	–	–	–	–
Aepycerotinae/*Nanger/Eudorcas/Raphicerus/Ourebia*	–	–	–	1	–	–	–	–	–	–	–	–
Aepycerotinae/Nanger/*Eudorcas/Raphicerus/Ourebia/Madoqua*/Reduncinae	–	–	–	2	–	–	–	–	–	–	–	–
Bovid 2	6	–	1	1	–	–	–	–	–	–	–	–
Bovid 3	4	–	1	1	–	–	–	–	–	–	–	–
Cf. Bovid 3	1	–	–	–	–	–	–	–	–	–	–	–
Leporidae	1	3	–	3	–	–	–	–	–	–	–	–
Bovid 2/3	–	–	1	–	–	–	–	–	–	–	–	–
Bovid ≥3	14	6	–	–	1	–	–	–	–	–	–	–
Rodentia	–		1	1	–	–	–	–	–	–	–	–
**Mammal size/indet**	**69**	**23**	**65**	**43**	**8**	**1**	**7**	**3**	**1**	**0**	**4**	**4**
Felidae/Canidae/*Loxodonta*/Mustelidae	–	–	–	1	–	–	–	–	–	–	–	–
Felidae/Mustelidae/Hyaenidae	–	1	–	–	–	–	–	–	–	–	–	–
Hypsodont ≥2	1	1	–	–	–	–	–	–	–	–	–	–
Hypsodont ≥3	8	1	1	1	–	–	–	–	1	–	–	–
Mammal 0.5	1	–	1	1	–	–	–	–	–	–	–	–
Mammal 1	3	–	5	3	–	–	–	–	–	–	–	–
Mammal 1 or 2	1	–	2	2	–	–	–	–	–	–	–	–
Mammal 2	7	2	5	3	1	–	–	–	–	–	1	1
Mammal 3	2	1	3	2	–	–	–	–	–	–	–	–
Mammal ≥3	22	10	–	–	3	–	–	–	–	–	–	–
Mammal ≥2	18	5	42	25	4	1	4	1	–	–	2	2
Mammal ≥1	5	2	3	2	–	–	2	1	–	–	1	1
Vertebrate indet.	1	–	3	3	–	–	1	1	–	–	–	–
**Total analyzed**	**102**	**102**	**69**	**69**	**9**	**9**	**7**	**7**	**1**	**1**	**4**	**4**
**NTAXA**	**3**	**10**	**1**	**5**	**1**	**3**	**0**	**1**	**0**	**1**	**0**	**0**

“Morph” indicates identifications using morphology only. “Morph + ZooMS” indicates identifications considering ZooMS results and morphology. Site codes: KAV, Kalundu Mound; KMP, SAL, Salumano; ZAM; Jakobo West Kapiri Mposhi; FIB, Fibobe II; MTS, Muteteshi.

The most common identification at the archaeological sites was to *Bos*, with 50 identifications, followed by Cephalophinae with 9 identifications. These include ZooMS-based identifications informed by specimen size (see below). Not all markers are needed for certain identifications. For example, the identification of Reduncinae does not require the COL1α2 757–789 (G/G’) markers if the COL1α2 978–990 (A/A’) markers are present. In some cases where necessary markers were not visible, specimen size could be used to narrow the identification: for example, when the COL1α2 757–789 (G/G’) marker was missing, size could be used to distinguish *Bos* from *Sylvicapra* or *Cephalophus* (Table 4; S2 Table). Some specimens had peptide markers that matched closely related species for which peptides are known. For example, one specimen each from Kalundu and Salumano had markers matching those published for suids [38]. Those markers also agree with the partial set identified for warthog (*Phacochoerus africanus*) by [47], and therefore this identification of suid is likely correct. Similarly, several fragments from small mammals had peptide markers matching those of leporids [112], and although not all peptide markers for members of the Leporidae are known, these identifications are likely correct, and morphology is consistent with Leporidae. Finally, one specimen also yielded good spectra, but was not attributable to any known taxon (Table 3; S2 Table) A wide range of wild fauna, including viverrids, rodents, and mustelids were recovered from earlier excavations at Kalundu [98]. Peptide markers for many of these African taxa have not been established and therefore further work on a comprehensive reference library is needed before they can be identified using ZooMS.

### Herding economies and the persistence of hunting in Iron Age Zambia

Despite uneven preservation and the often coarse nature of ZooMS-based identifications, the results of our analysis provide insights into the Iron Age economies of southwestern and central Zambia. Cattle were identified at every site except for Muteteshi, which had a small sample size (n = 4) and exceptionally poor collagen preservation. Caprines were only identified at Kalundu—evidenced by a single specimen. Kalundu, Kapiri Mposhi, and Salumano also yielded evidence for wild bovids, but the three smallest assemblages did not. The wild bovid assemblage at Kalundu is largely comprised of duikers, but these were not identified at Kapiri Mposhi, although based on partial spectra, one reduncine and several less identifiable wild bovids were recognized in the assemblage. The Kalundu assemblage also yielded the largest diversity of wild fauna, including Hippotragine, Tragelaphine, and Antilopine bovids, as well as other large mammals, such as suid, elephant, and rhinoceros. The ZooMS-based identifications from the sites analyzed here align well with reported faunal remains from previous excavations at these and other Iron Age sites in Zambia.

Analysis of fauna from previous excavations at Salumano (c. 2300 BP) reveal cattle in early horizons of the site, with caprines appearing later [116]. Similarly, cattle are the only domesticate present in the earlier component at Nanga (c. 1240 to 1070 BP) [101, 137], with cattle and caprines occurring together only in the later component of the site (c. 980 BP) [137]. These patterns point to a broadening of pastoral economies over the course of the Iron Age. Earlier excavations at Kalundu, as well as Isamu Pati, another Kalomo culture site, revealed that diverse wild fauna, particularly duiker, comprised a major portion of the assemblages [98]. Both Kalundu and Isamu Pati assemblages yielded cattle remains, as well as cattle figurines. Caprine remains are also present at both sites—cattle and caprine mandibles are in equal number at Isamu Pati, though no quantification data are given for the Kalundu caprines. Identifiable fauna from Iron Age levels at Makwe in eastern Zambia also show a predominance of cattle, in addition to wild fauna, primarily suids [100]. Identifiable fauna from the Early Iron Age site of Namakala (c.1400 BP) revealed only wild species [101, 138]. The continued reliance on wild fauna has been attributed to the constant threat of trypanosomiasis, communicated by the tsetse fly (*Glossina* spp.), which is abundant across southern and southeastern Zambia [139]. Caprines, particularly goats, are able to avoid tsetse fly blood meals much more effectively than are cattle [140]. Goats are mixed feeders that prefer to browse, and may be used to suppress brush, particularly in tsetse-ridden environments [141, 142]. Although sample sizes are small, the abundance of cattle and relative paucity of goat at Kalundu and Kapiri Mposhi hint at a relatively tsetse-free environment. Our analyses thus point to a unique pattern of cattle-based pastoralism supplemented with hunting, particularly duikers, as evidenced at Kalundu. These wild bovids would have provided an abundant source of meat—even intensive hunting as part of tsetse control efforts in the 1960s did not reduce duiker populations in eastern Zambia [143].

## Conclusion

With the development of a reference set of peptide markers for African bovids, we demonstrate that identification to subfamily, and sometimes narrower taxonomic categories, is possible through ZooMS. By incorporating ZooMS into zooarchaeological analyses, the contours of foodways in Iron Age Zambia become more clear. Results show that in central and southwestern Zambia, Iron Age subsistence economies and foodways were based largely on cattle, but, as seen at Kalundu, wild bovids were also important. Further analyses of larger assemblages are necessary to reveal more detailed information on herding economies, culinary processing techniques, and foodways.

More broadly, identification of fragmentary material to subfamily and tribe is incredibly useful, given the dietary and habitat requirements of these different bovid groups. Members of Alcelaphinae are grassland-dwelling animals, whereas many species of Cephalophinae are typically found in more forested, closed-canopy environments. Such identifications can provide some insight into past environments, and provide identifications that can inform further analyses, including stable isotope analysis. In some cases, genus-level identifications are possible for wild bovids such as the klipspringer, dikdik, and suni. Such specific identifications provide avenues for assessing deeper questions regarding shifts in climate, environment, as well as hunting and land-use pressures that may have contributed to changes in faunal distributions over time in a variety of African contexts.

Different bovid groups share many peptide markers with identical amino acid sequences, and thus m/z ratios, secure taxonomic identifications can typically only be made on specimens with well-preserved collagen and full spectra. Because size class can sometimes help narrow taxonomic identifications, we recommend that, if available, specimens identifiable to element and size be analyzed for ZooMS. Furthermore, while this study includes species from all bovid tribes, out results point to considerable diversity in peptide markers within the Antilopinae, and some differences in the new markers exist within subfamilies or tribes. Further analyses of bovids within some subfamilies are necessary to clarify these differences.

Our results demonstrate the applicability of ZooMS for the study of even very highly fragmented and poorly preserved assemblages in African archaeological contexts. Using ZooMS, domesticates, including cattle, sheep, and goat, can usually be distinguished from most wild African bovid species, and therefore this method will be extremely useful in investigations of the dispersal of these domestic livestock across the African continent. And though ZooMS-based identifications are restricted to broader taxonomic categories in most cases, the method will still be of great benefit in the analysis of very fragmentary and poorly preserved assemblages, and can provide greater consistency in identifications among analysts. ZooMS certainly does not render zooarchaeology obsolete of course; identification of African bovids through this method instead opens up more possibilities for integrating this method with standard zooarchaeological analyses, allowing for deeper analyses of taxon-specific differences in transport, carcass handling, and culinary processing [2, 144], as well as taphonomic processes, which contribute to major identification biases [145–147]. Recent work elsewhere has shown the usefulness of ZooMS in assessing differential processing strategies [110]. Application of ZooMS on a large scale has great potential for clarifying taxon-specific patterns of preservation.

## Supporting information

S1 Fig MALDI-TOF spectra from the bovids. The following pages contain one example spectra from each of the species used in the study. Each subfamily has a page with grouped spectra and then each individual has a peak picked spectra individually. The first spectra in the grouped spectra for each subfamily is always *Bos taurus* (Bovinae) for easy comparison to a previously published species.
(PDF)

S2 Fig MS/MS sequence identification of biomarkers. The following pages show the MS/MS spectra of the peptides at each of the diagnostic biomarkers presented in the manuscript. All images from Byonic.
(PDF)

S3 Fig MS/MS sequence identification of the peptides at m/z 3017. In Bovidae the peak at m/z 3017 is composed of two peptides. One peptide is diagnostic between different species (COL1α2 757–789 or G). The other peptide is shared between all bovids and therefore non-diagnostic (COL1α2 89–130). This is why m/z 3017 should not be used for taxonomic identification. The diagnostic peptide is the only peptide which composes the MALDI peak with a different number of oxidations (m/z 3033) and therefore that is diagnostic. Images from Byonic.
(PDF)

S1 Table Modern species which were collected for reference data used in this study along with their numbers. OM numbers are the specimen numbers for the samples we collected, which are housed in the Osteology Section at the National Museums of Kenya in Nairobi. Samples in bold were analyzed by LC-MS/MS.
(XLSX)

S2 Table Archaeological data. ZooMS & Morphology ID takes into account morphological indicators, such as body size, to refine the ZooMS-based identification. Site codes: KAV, Kalundu Mound; KMP, SAL, Salumano; ZAM; Jakobo West Kapiri Mposhi; FIB, Fibobe II; MTS, Muteteshi.
(XLSX)

S3 Table MS/MS information. Table shows the numbers for the LC-MS/MS samples as well as summary statistics from the runs.
(XLSX)

S4 Table List of all top proteins from the search against SwissProt. A total of 168 proteins were found in at least one sample and were then added to the collagen sequence database for the marker authentication.
(XLSX)

S5 Table Marker list for all species including sequences, masses, and numbers of oxidations. Masses in light italics are present in the LC-MS/MS, but are not suitable for species identification using MALDI because they are present in more than one marker or those masses are also the masses of common contaminants. Boviane—Tragaelaphini—Group1: *Taurotragus*, *Tragelaphus buxtoni*; *Tragelaphus eurycerus*; Boviane—Tragaelaphini—Group2: *Tragelaphus imberis*, *Tragelaphus strepsiceros*, *Tragelaphus scriptus*, *Tragelaphus spekii*. *Predicted from the genetic sequence data only, not confirmed with MALDI data. **The both the fully cleaved peptide and the peptide with one missed cleavage are present in the LC-MS/MS and the MALDI so are presented here as a joint marker. ***Previously published species. ****New markers presented in this paper. 1588 for Neotraginae is in the trailing peaks of another peptide at 1586.
(XLSX)

S1 File Information on data accessibility, nomenclature of ZooMS markers, and a note on *Neotragus pygmaeus*. (PDF)
