# Supplementary material for: Distinguishing African bovids using Zooarchaeology by Mass Spectrometry (ZooMS): New peptide markers and insights into Iron Age economies in Zambia
Source: PLoS One. 2021 May 18;16(5):e0251061. doi: 10.1371/journal.pone.0251061 (PMC8130928; doi:10.1371/journal.pone.0251061)
Supplement: S1 Fig — The following pages contain one example spectra from each of the species used in the study. Each subfamily has a page with grouped spectra and then each individual has a peak picked spectra individually. The first spectra in the grouped spectra for each subfamily is always Bos taurus (Bovinae) for easy comparison to a previously published species. (PDF) [file pone.0251061.s001.pdf]

S1 Fig: MALDI-TOF Spectra from the Bovids

The following pages contain one example spectra from each of the species used in the study. Each subfamily has a page with grouped spectra and then each individual has a peak picked spectra individually. The first spectra in the grouped spectra for each subfamily is always *Bos taurus* (Bovinae) for easy comparison to a previously published species.

Subfamily Bovinae

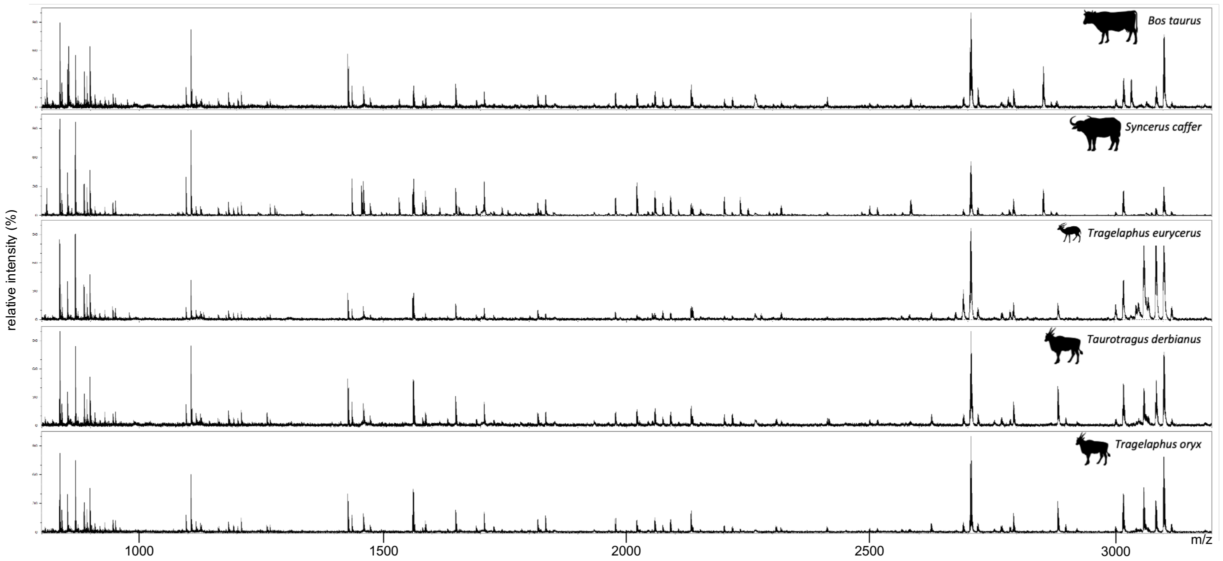

Subfamilies Hippotraginae and Reduncinae

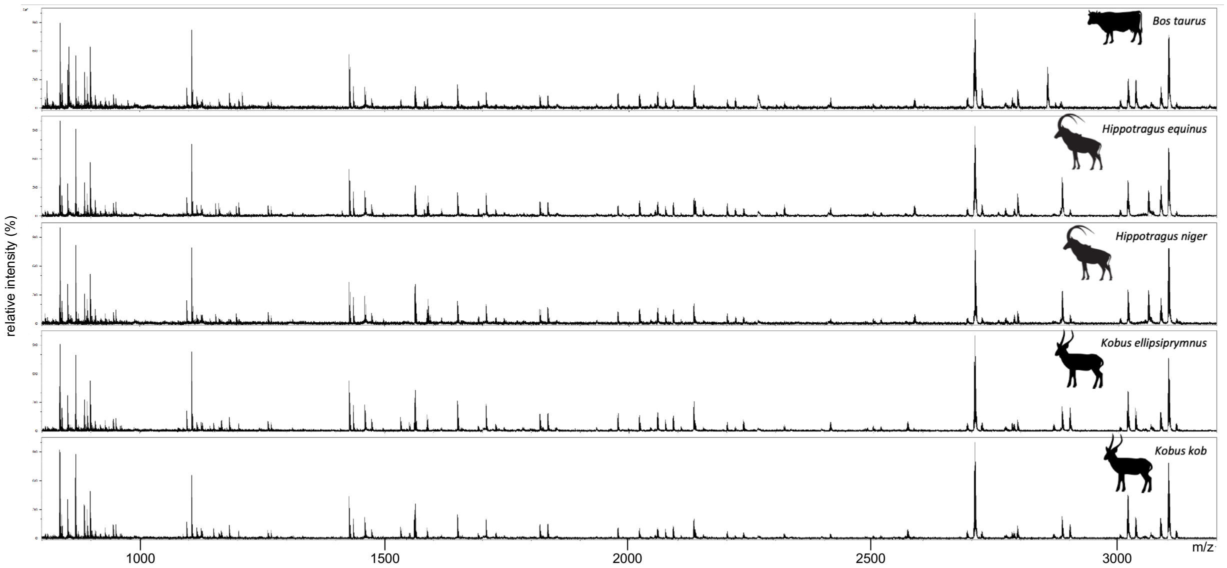

Subfamily Alcelaphinae

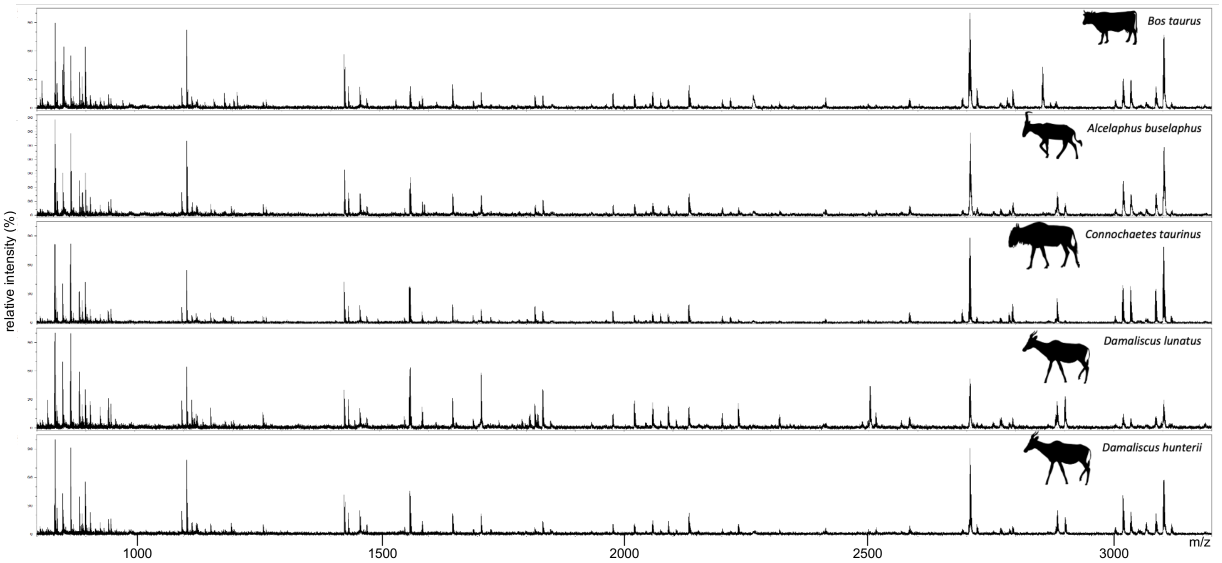

Subfamily Cephalophinae

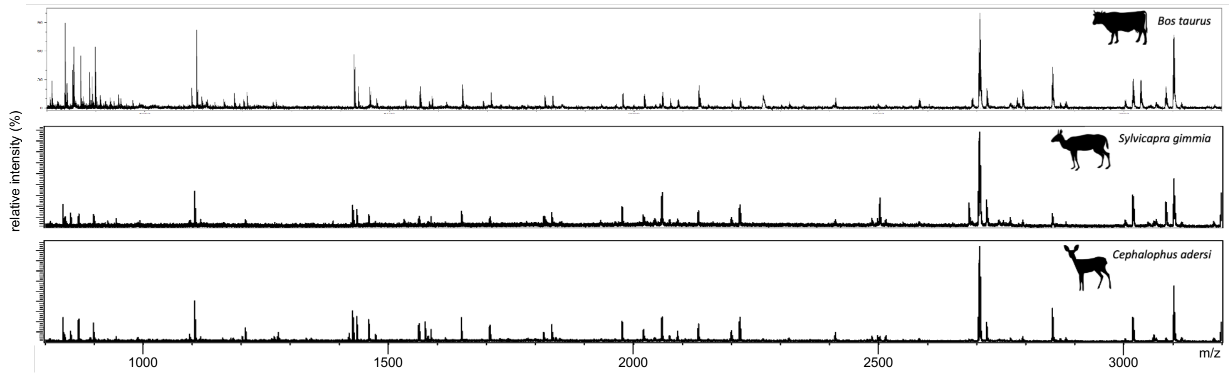

Subfamily Antilopinae

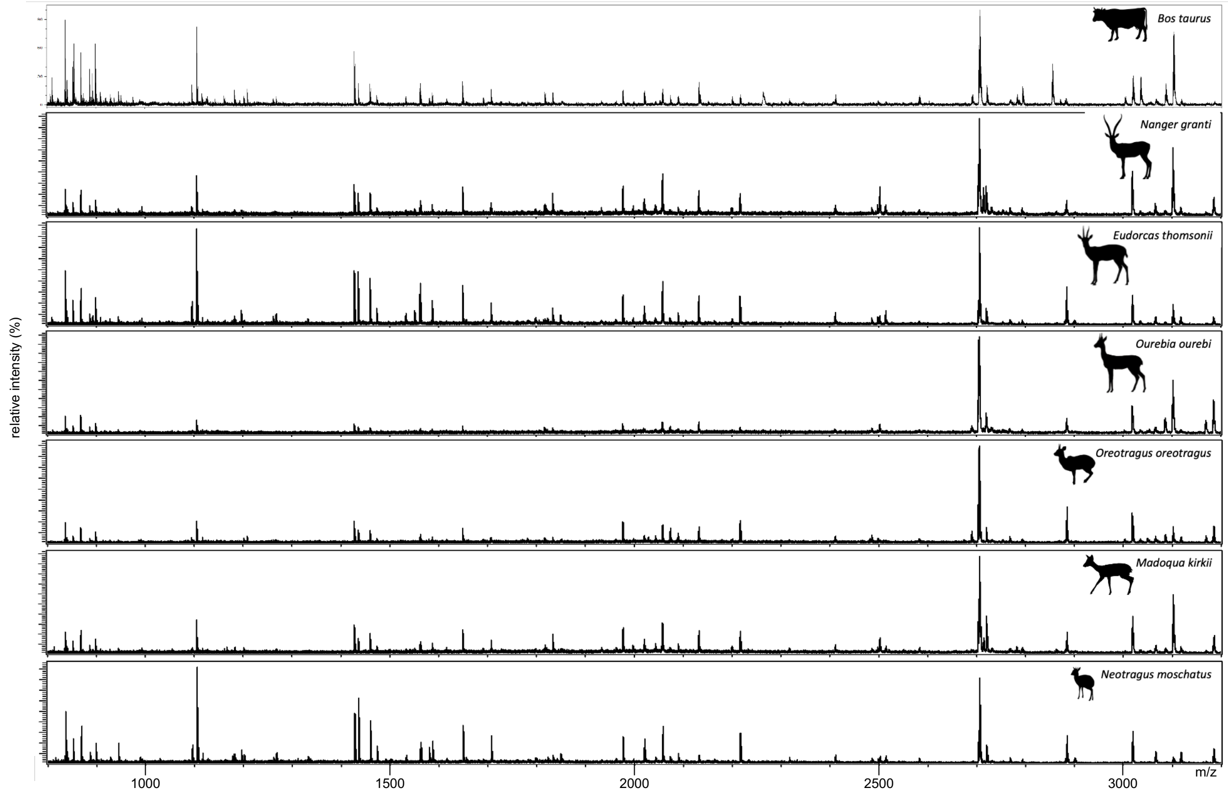

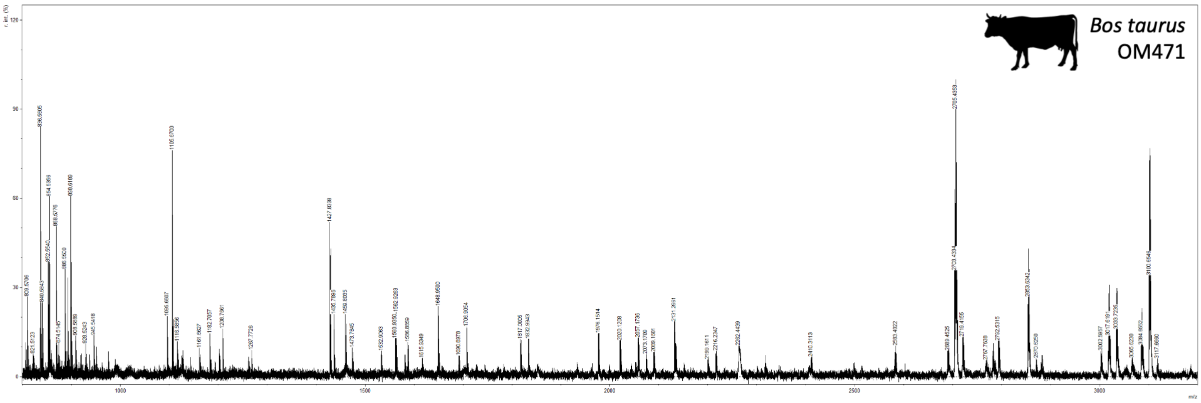
Bos taurus
OM471

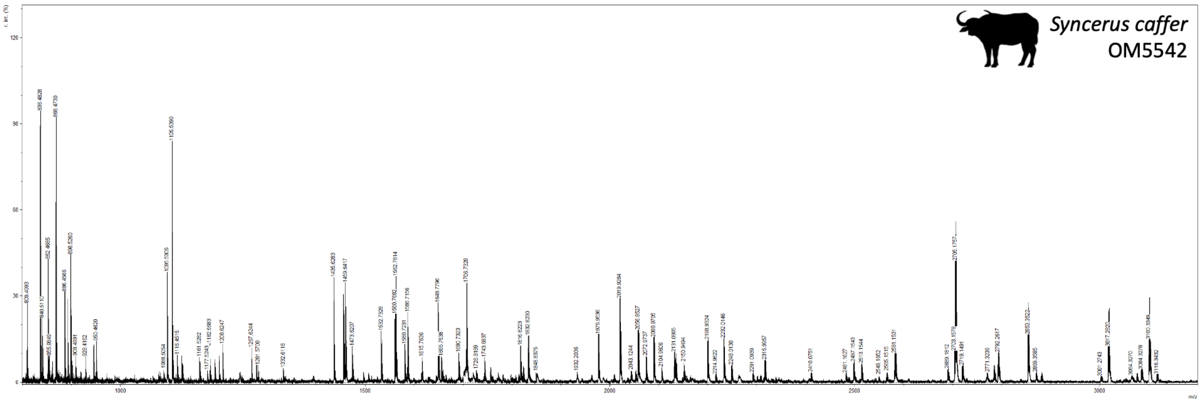
Syncerus caffer
OM5542

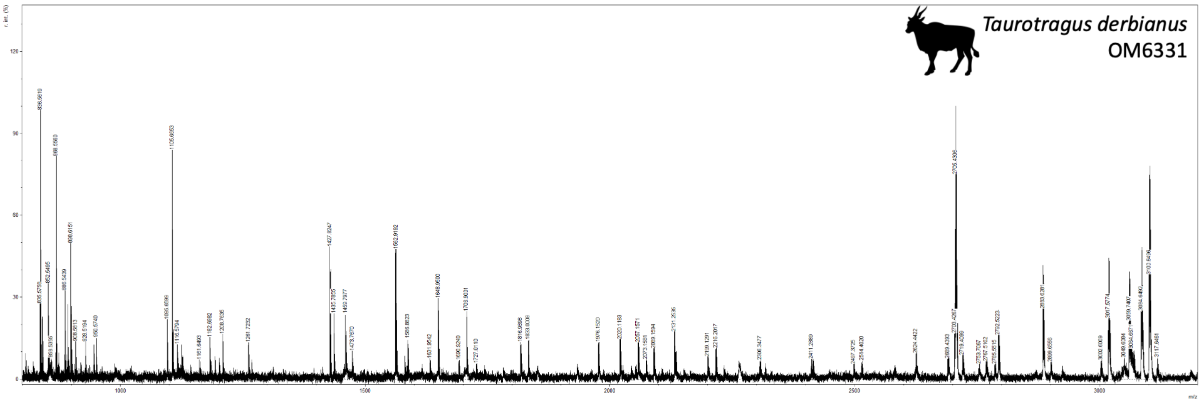
Taurotragus derbianus
OM6331

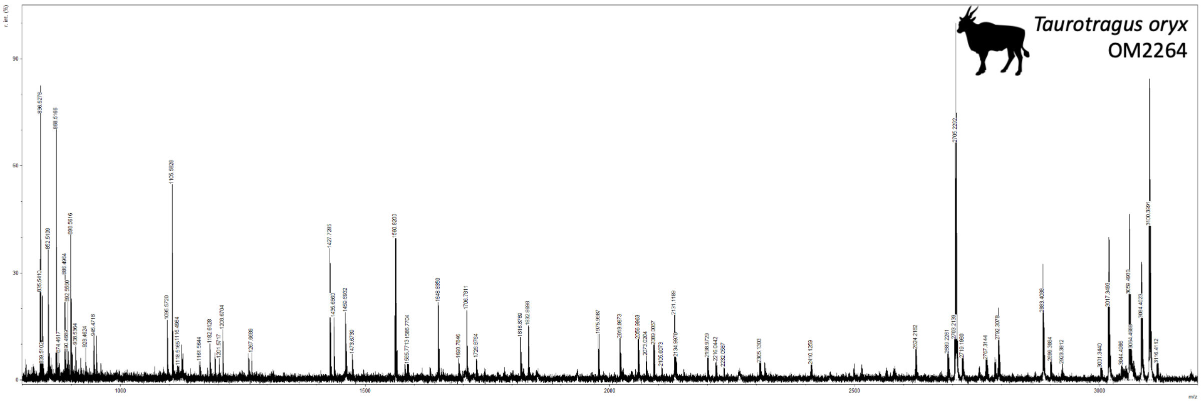
Taurotragus oryx
OM2264

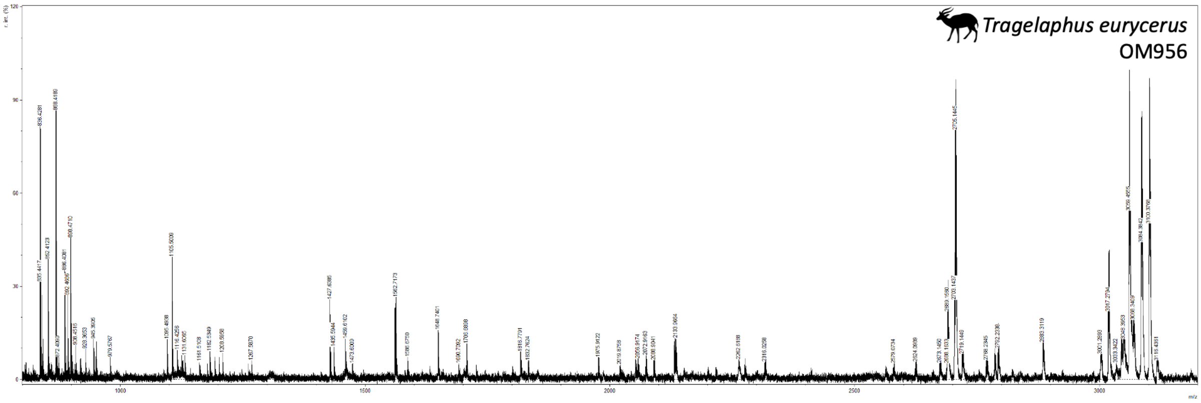
Tragelaphus eurycerus
OM956

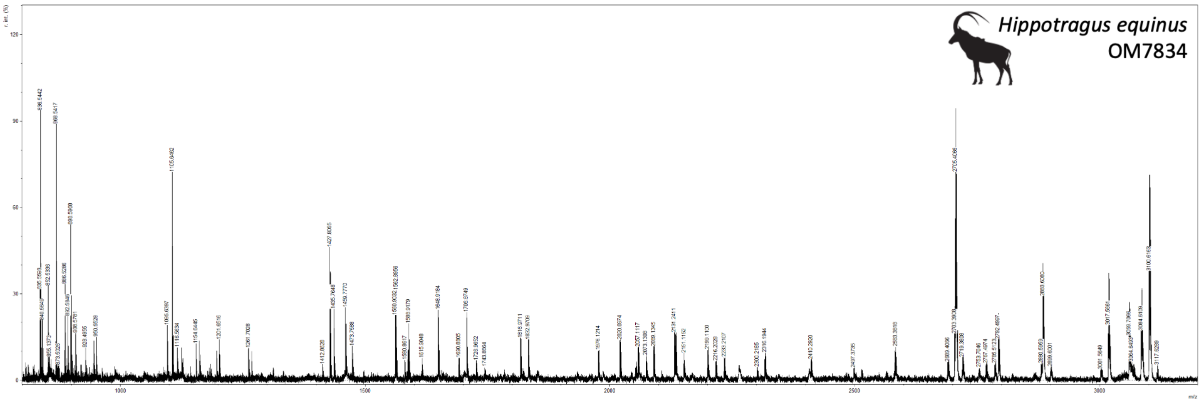
Hippotragus equinus
OM7834

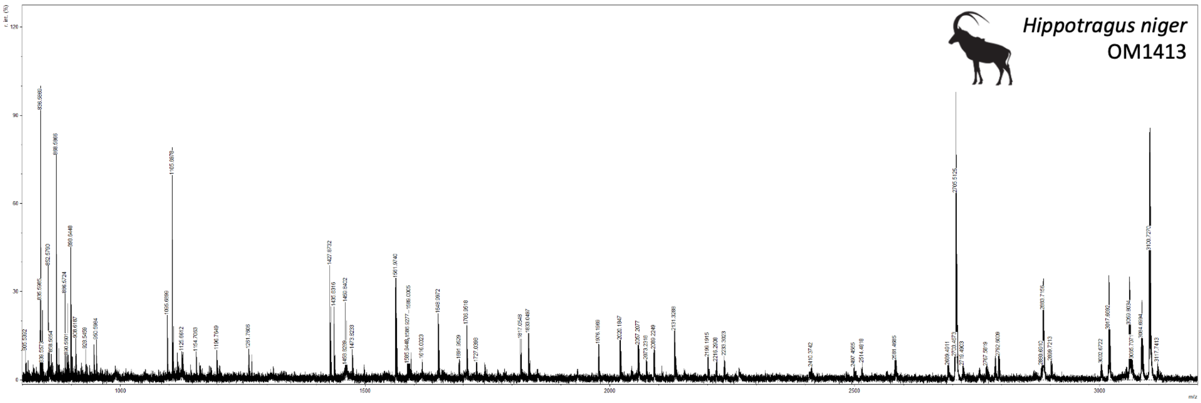
Hippotragus niger
OM1413

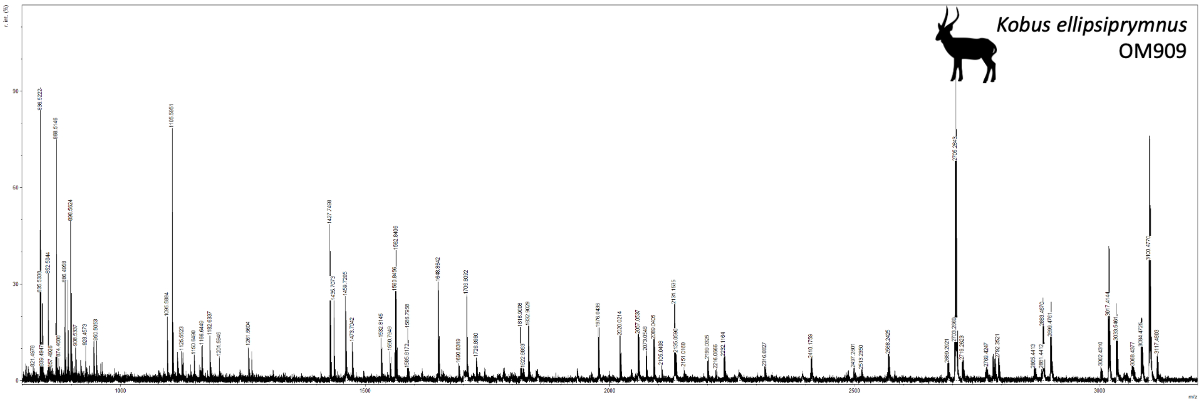
Kobus ellipsiprymnus
OM909

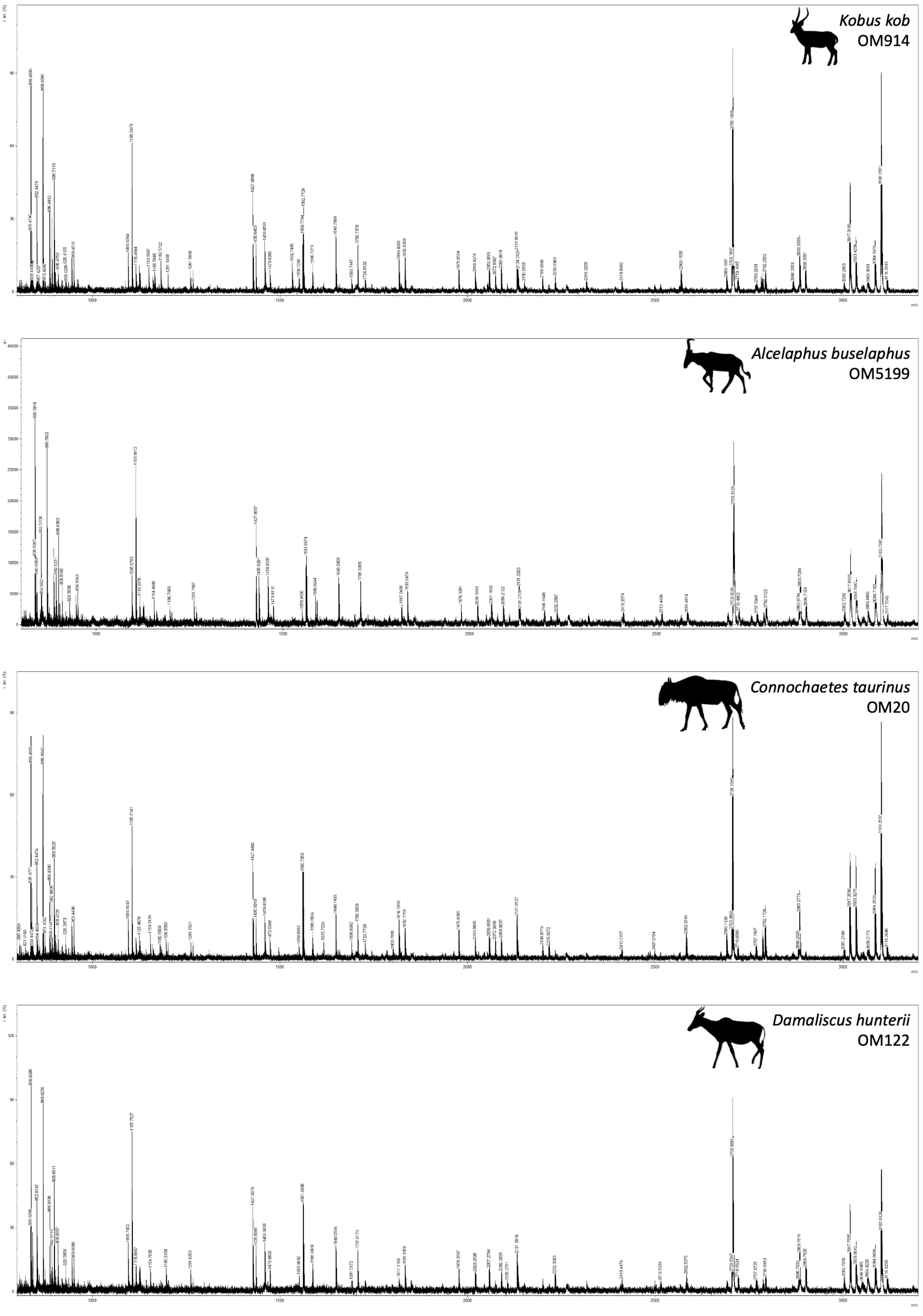
Kobus kob
OM914
Alcelaphus buselaphus
OM5199
Connochaetes taurinus
OM20
Damaliscus hunterii
OM122

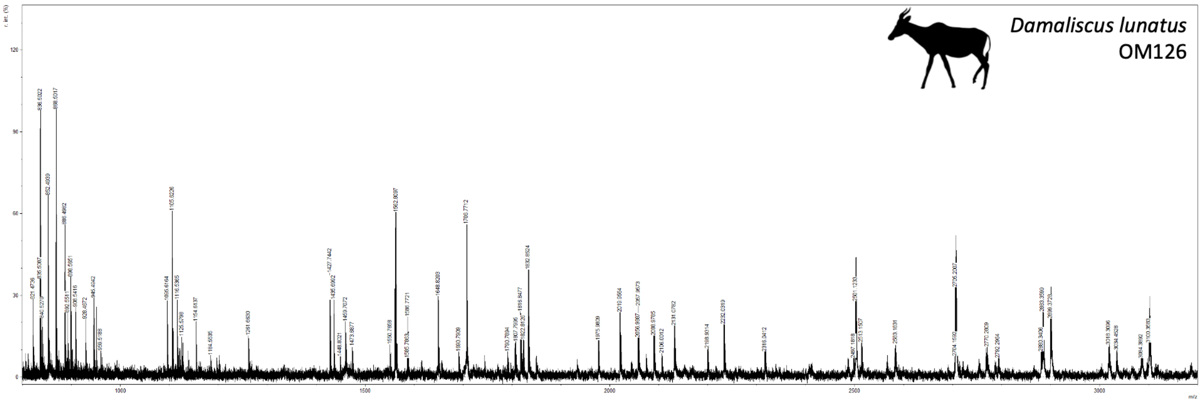
Damaliscus lunatus
OM126
m/z

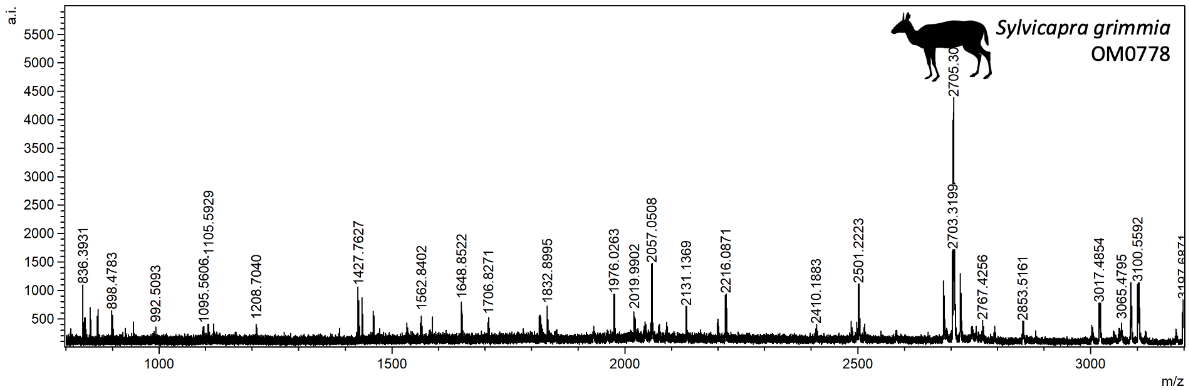
Sylvicapra grimmia
OM0778
a.i.
m/z
836.3931
898.4783
992.5093
1095.5606
1105.5929
1208.7040
1427.7627
1562.8402
1648.8522
1706.8271
1832.8995
1976.0263
2019.9902
2057.0508
2131.1369
2216.0871
2410.1883
2501.2223
2703.3199
2705.30
2767.4256
2853.5161
3017.4854
3065.4795
3100.5592

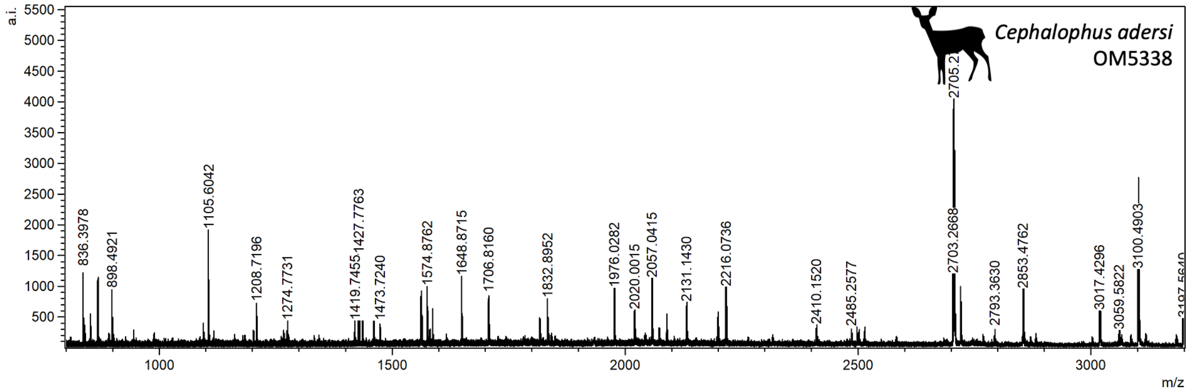
Cephalophus adersi
OM5338
a.i.
m/z
836.3978
898.4921
1105.6042
1208.7196
1274.7731
1419.7455
1427.7763
1473.7240
1574.8762
1648.8715
1706.8160
1832.8952
1976.0282
2020.0015
2057.0415
2131.1430
2216.0736
2410.1520
2485.2577
2703.2668
2705.2
2793.3630
2853.4762
3017.4296
3059.5822
3100.4903

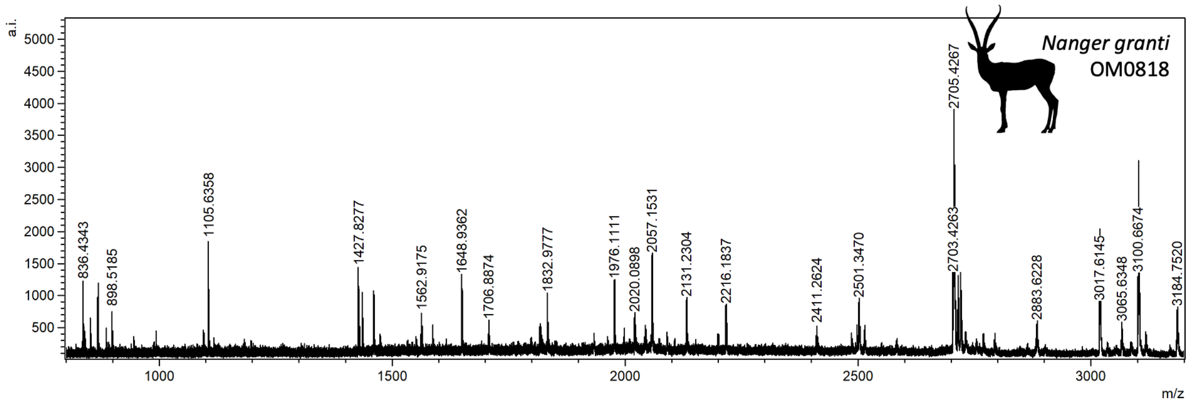
Nanger granti
OM0818
a.i.
m/z
836.4343
898.5185
1105.6358
1427.8277
1562.9175
1648.9362
1706.8874
1832.9777
1976.1111
2020.0898
2057.1531
2131.2304
2216.1837
2411.2624
2501.3470
2703.4263
2705.4267
2883.6228
3017.6145
3065.6348
3100.6674
3184.7520

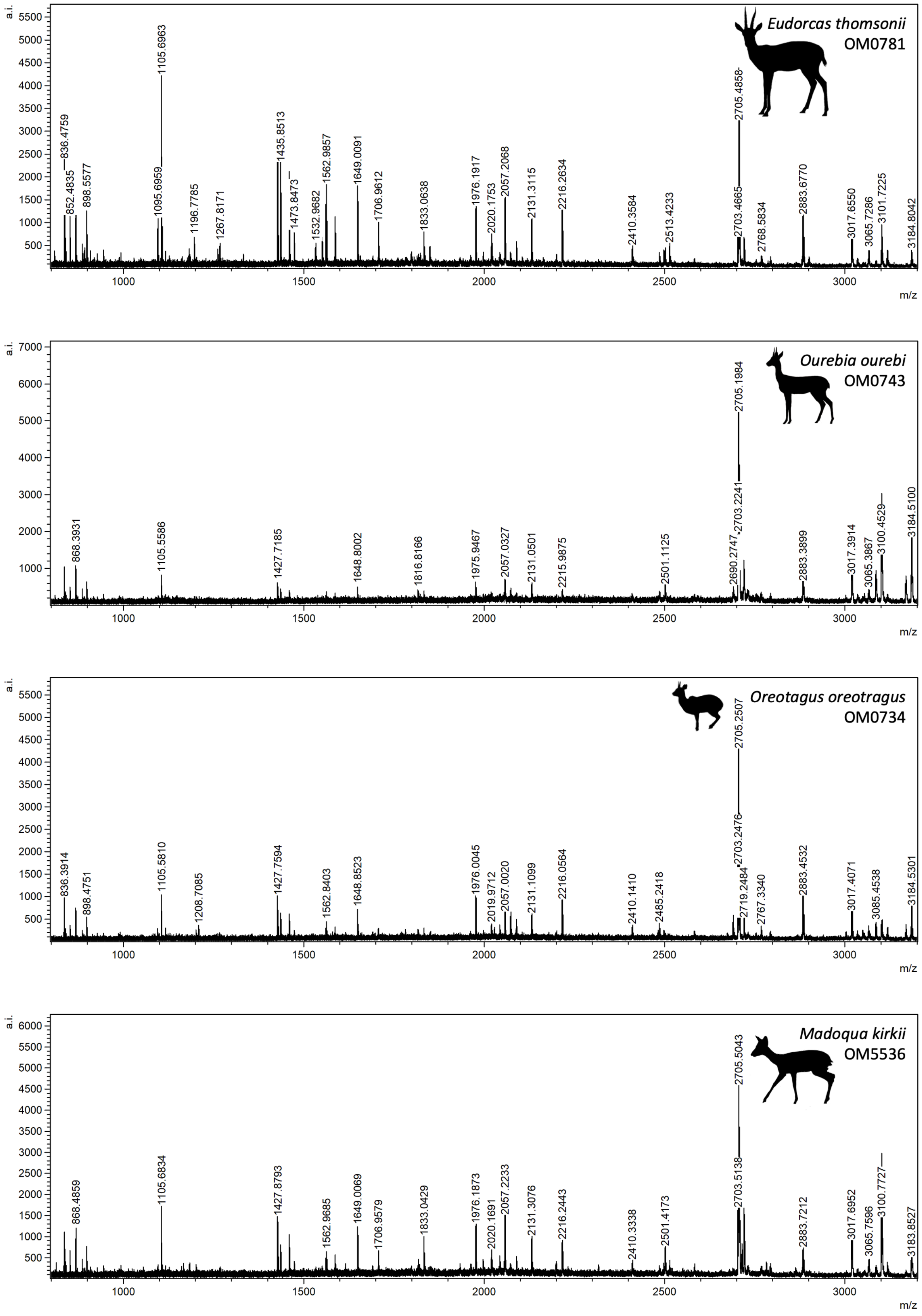
Eudorcas thomsonii
OM0781
Ourebia ourebi
OM0743
Oreotagus oreotragus
OM0734
Madoqua kirkii
OM5536
a.i.
m/z
836.4759
852.4835
898.5577
1095.6959
1105.6963
1196.7785
1267.8171
1435.8513
1473.8473
1532.9682
1562.9857
1649.0091
1706.9612
1833.0638
1976.1917
2020.1753
2057.2068
2131.3115
2216.2634
2410.3584
2513.4233
2703.4665
2705.4858
2768.5834
2883.6770
3017.6550
3065.7286
3101.7225
3184.8042
868.3931
1105.5586
1427.7185
1648.8002
1816.8166
1975.9467
2057.0327
2131.0501
2215.9875
2501.1125
2690.2747
2703.2241
2705.1984
2883.3899
3017.3914
3065.3867
3100.4529
3184.5100
836.3914
898.4751
1105.5810
1208.7085
1427.7594
1562.8403
1648.8523
1976.0045
2019.9712
2057.0020
2131.1099
2216.0564
2410.1410
2485.2418
2703.2476
2705.2507
2719.2484
2767.3340
2883.4532
3017.4071
3085.4538
3184.5301
868.4859
1105.6834
1427.8793
1562.9685
1649.0069
1706.9579
1833.0429
1976.1873
2020.1691
2057.2233
2131.3076
2216.2443
2410.3338
2501.4173
2703.5138
2705.5043
2883.7212
3017.6952
3065.7596
3100.7727
3183.8527

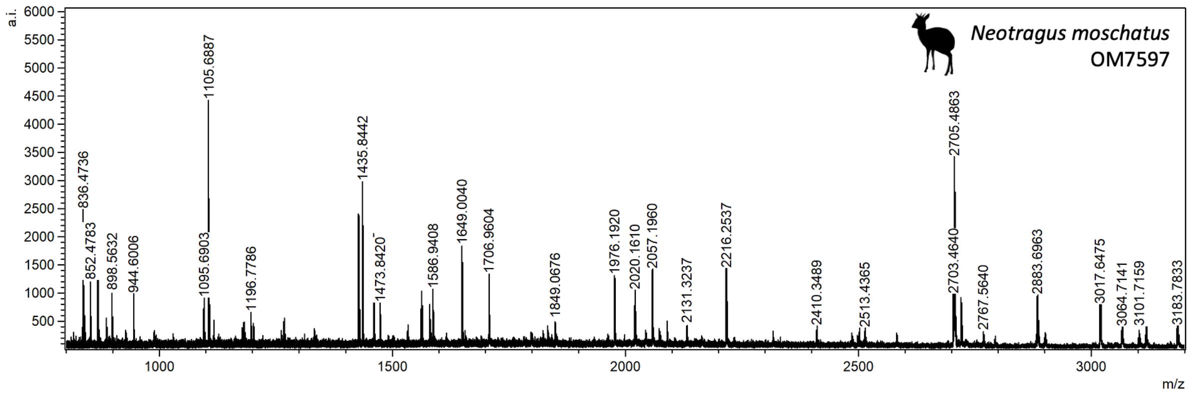

Neotragus moschatus
OM7597
a.i.
m/z
836.4736
852.4783
898.5632
944.6006
1095.6903
1105.6887
1196.7786
1435.8442
1473.8420
1586.9408
1649.0040
1706.9604
1849.0676
1976.1920
2020.1610
2057.1960
2131.3237
2216.2537
2410.3489
2513.4365
2703.4640
2705.4863
2767.5640
2883.6963
3017.6475
3064.7141
3101.7159
3183.7833
