# Supplementary material for: Distinguishing African bovids using Zooarchaeology by Mass Spectrometry (ZooMS): New peptide markers and insights into Iron Age economies in Zambia
Source: PLoS One. 2021 May 18;16(5):e0251061. doi: 10.1371/journal.pone.0251061 (PMC8130928; doi:10.1371/journal.pone.0251061)

S2 Fig. MS/MS Sequence Identification of Biomarkers

The following pages show the MS/MS spectra of the peptides at each of the diagnostic biomarkers presented in the manuscript. All images from Byonic.

COL1α1 586 – 618 (F)

m/z 2853

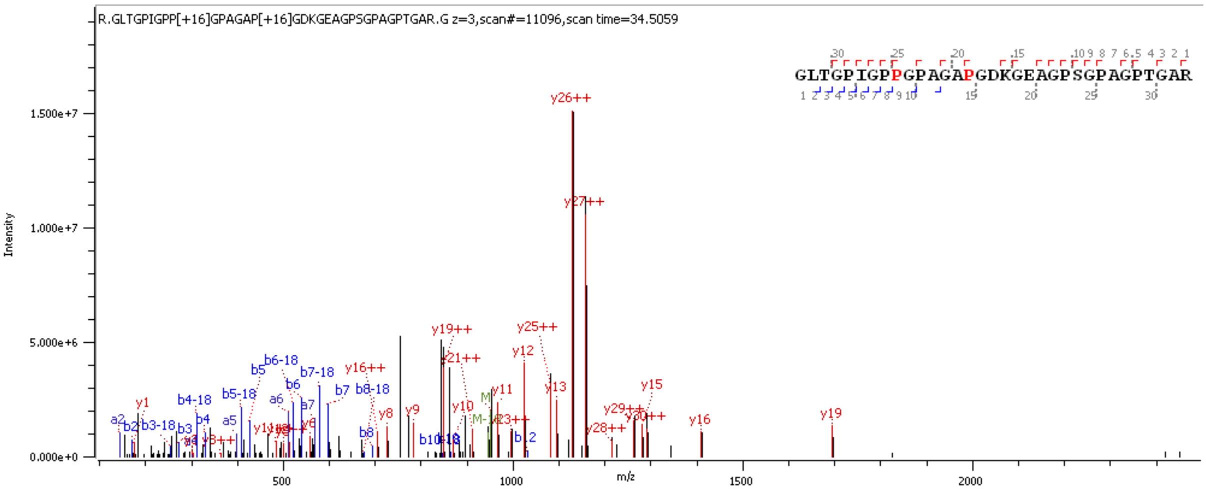

m/z 2883

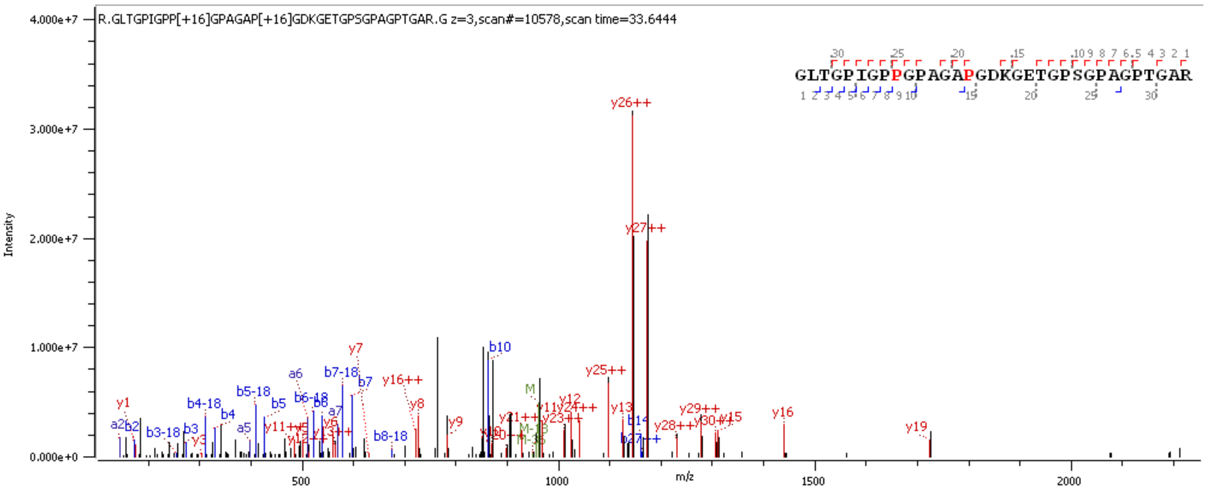

COL1α2 375 – 386 (novel marker)

m/z 1154

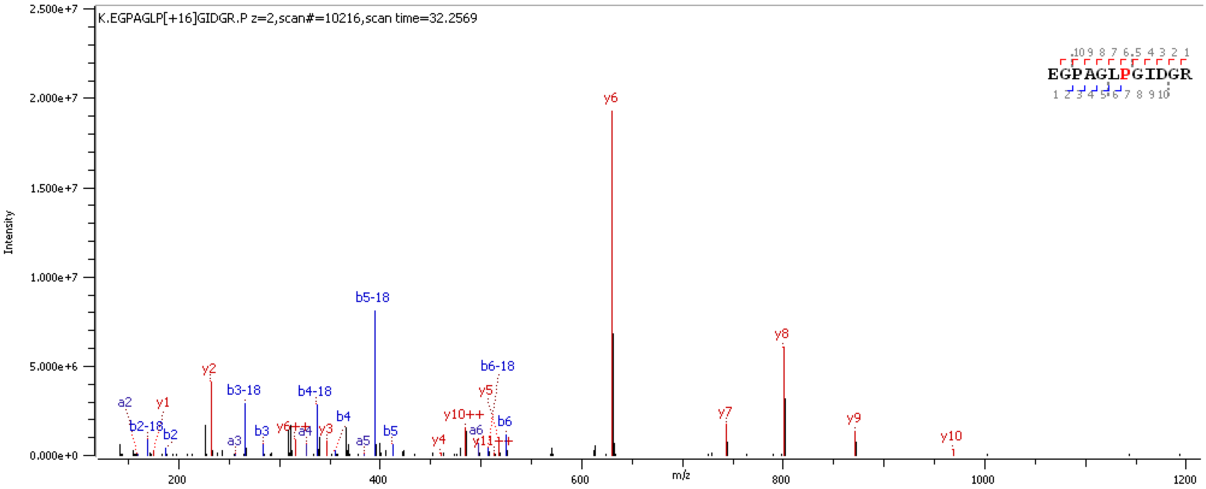

m/z 1182

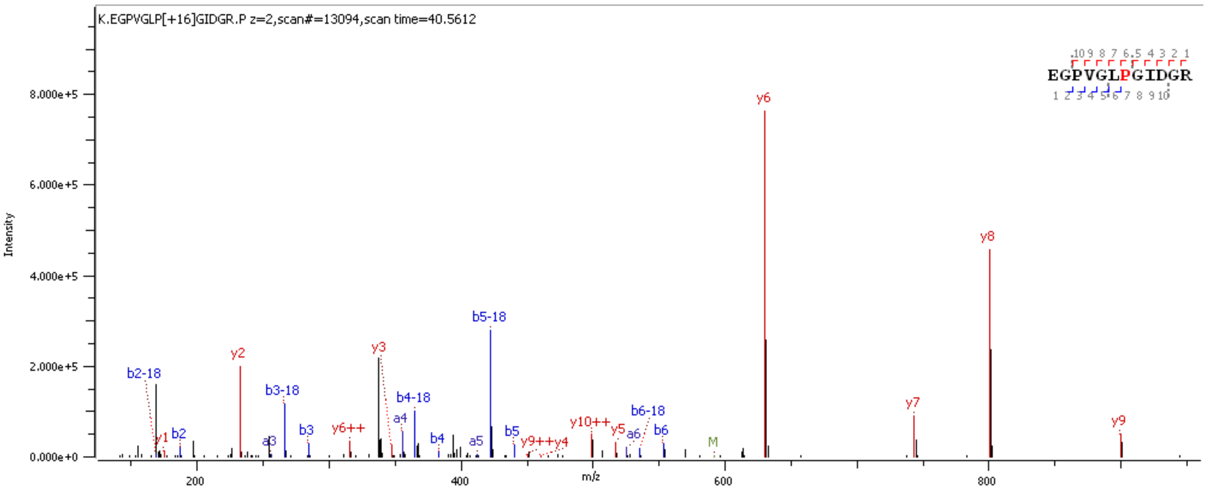

COL1α2 484 – 498 (B)

m/z 1427

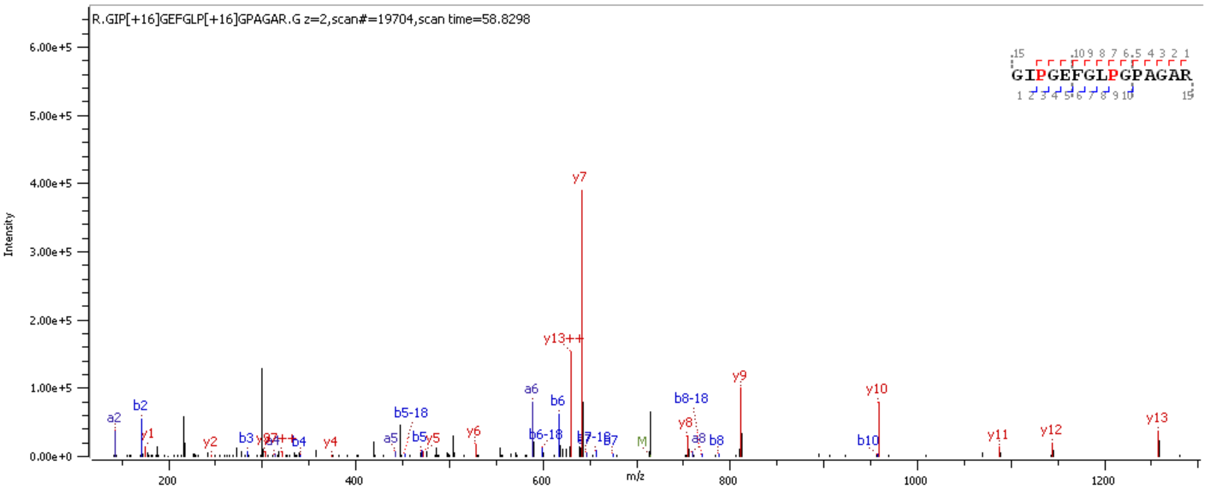

m/z 1455

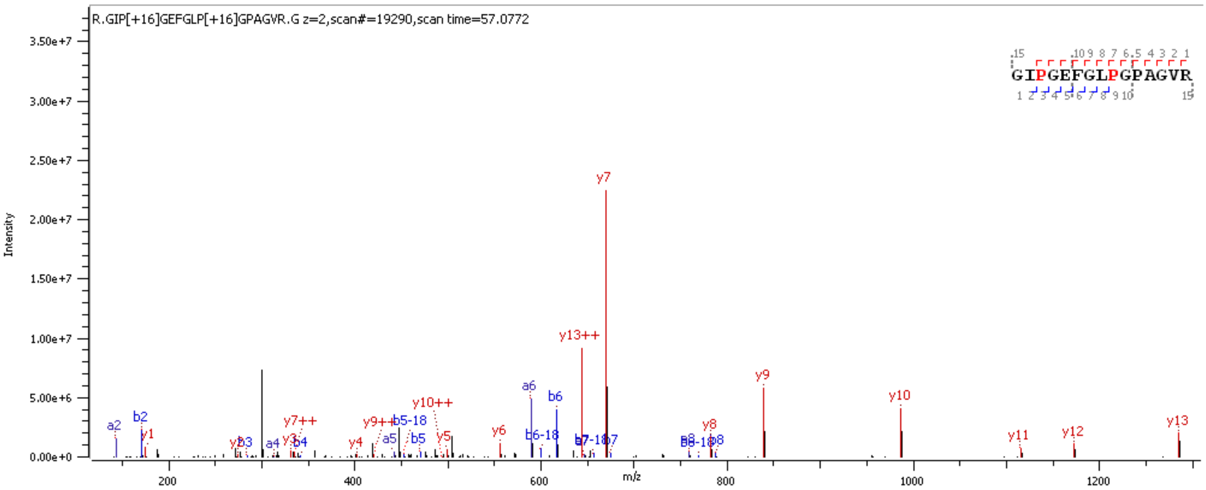

COL1α2 502 - 519 (C)

m/z 1550

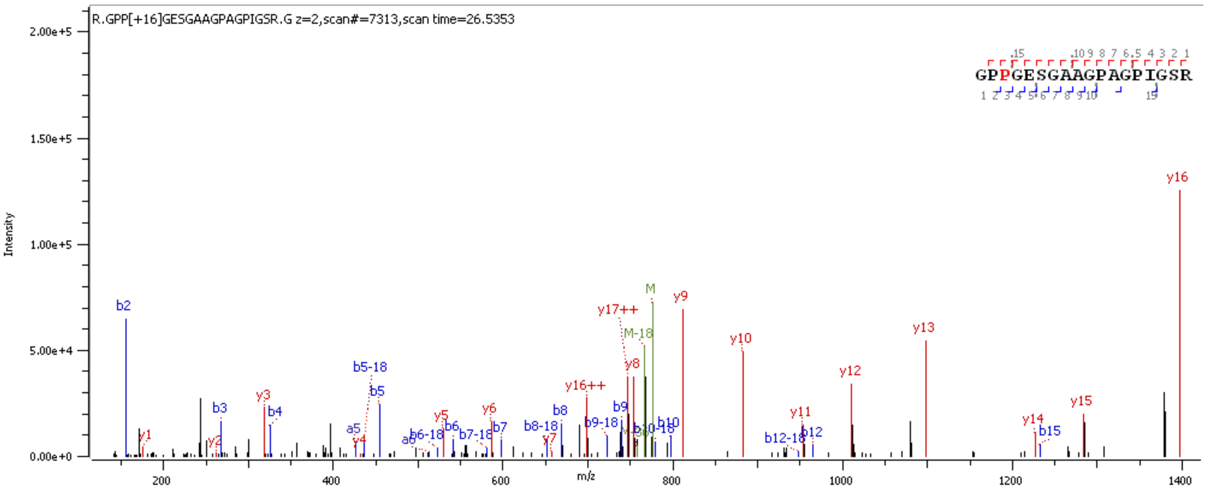

m/z 1580

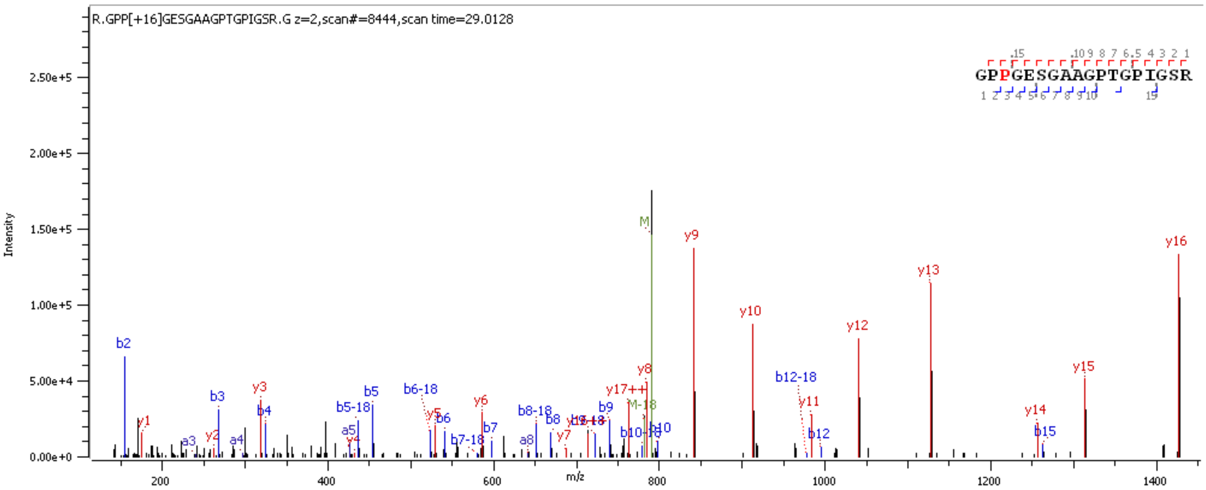

COL1α2 757 – 789 (G)

m/z 3033

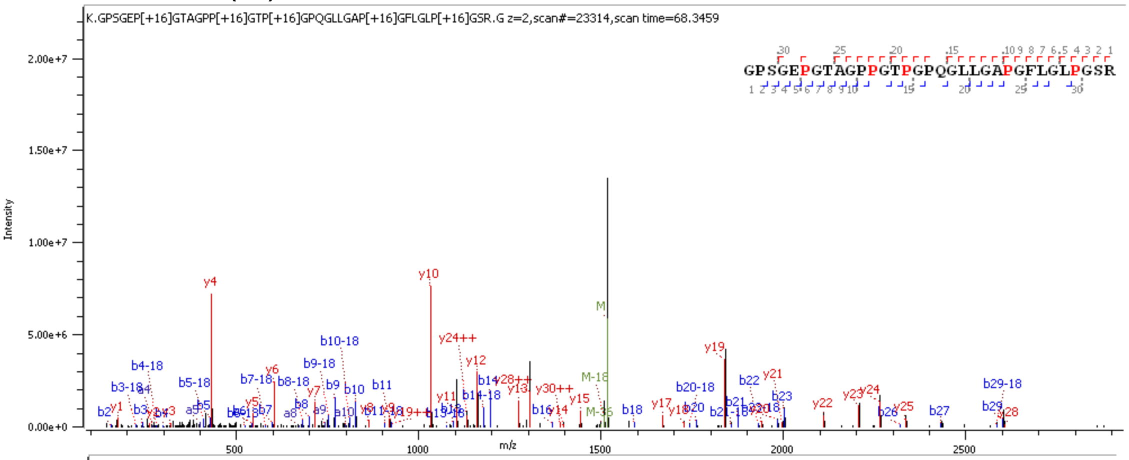

m/z 3043

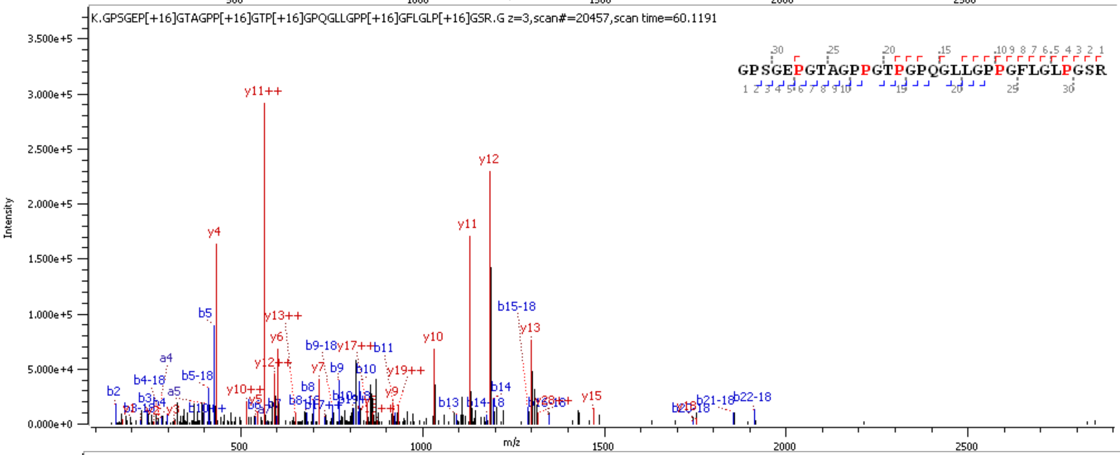

m/z 3059

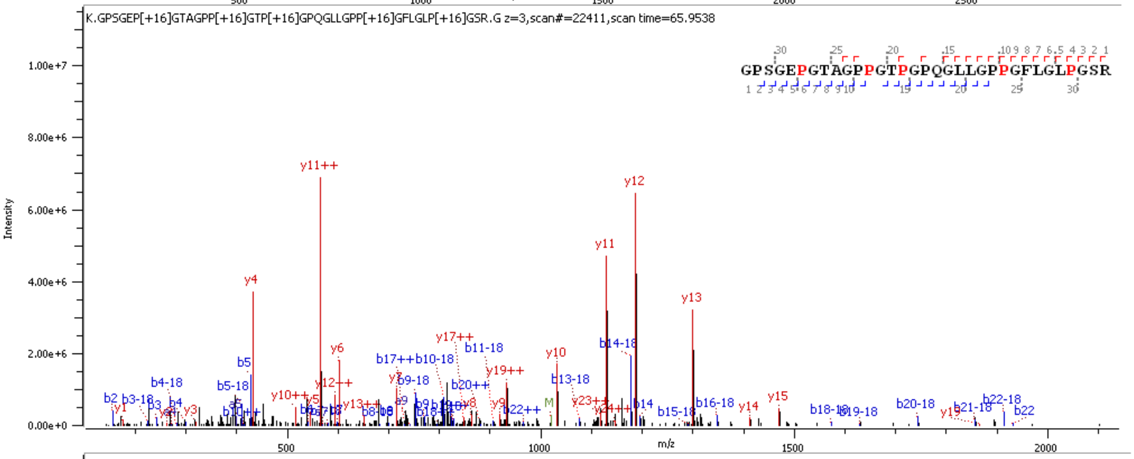

m/z 3075

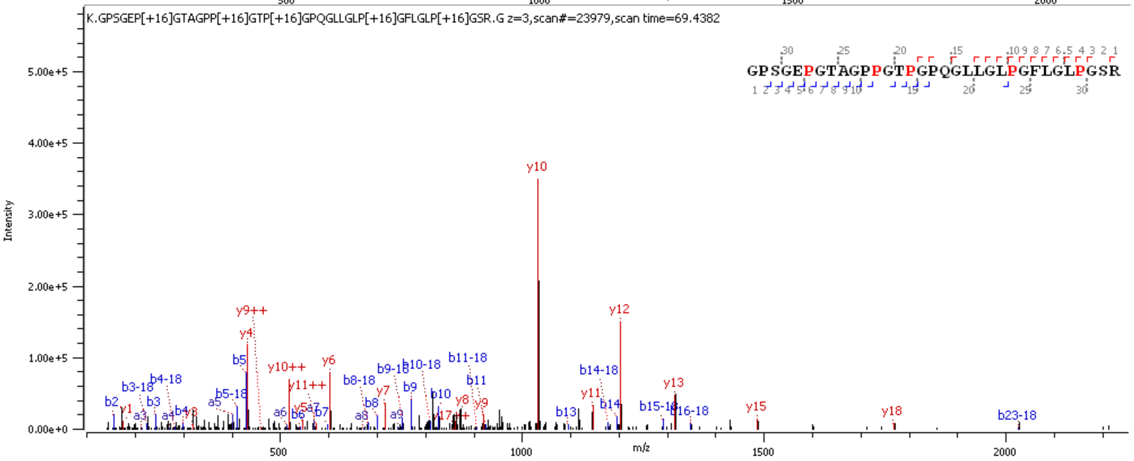

COL1α2 889 – 906 (novel marker)

m/z
1532

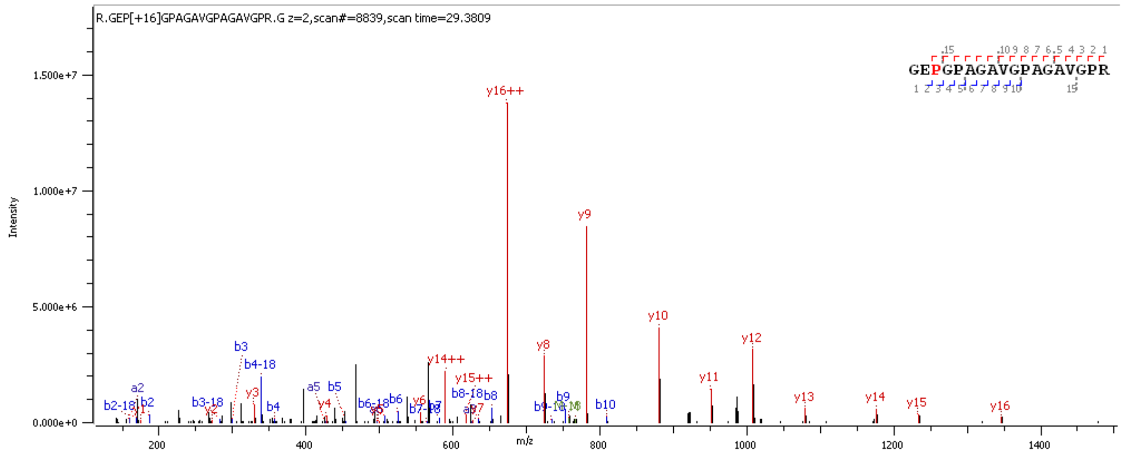

m/z
1574

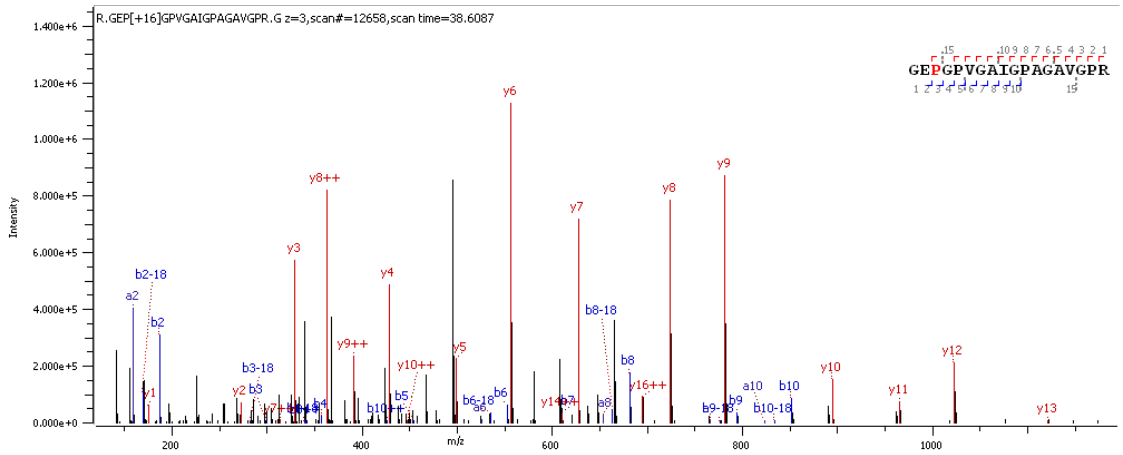

m/z
1588

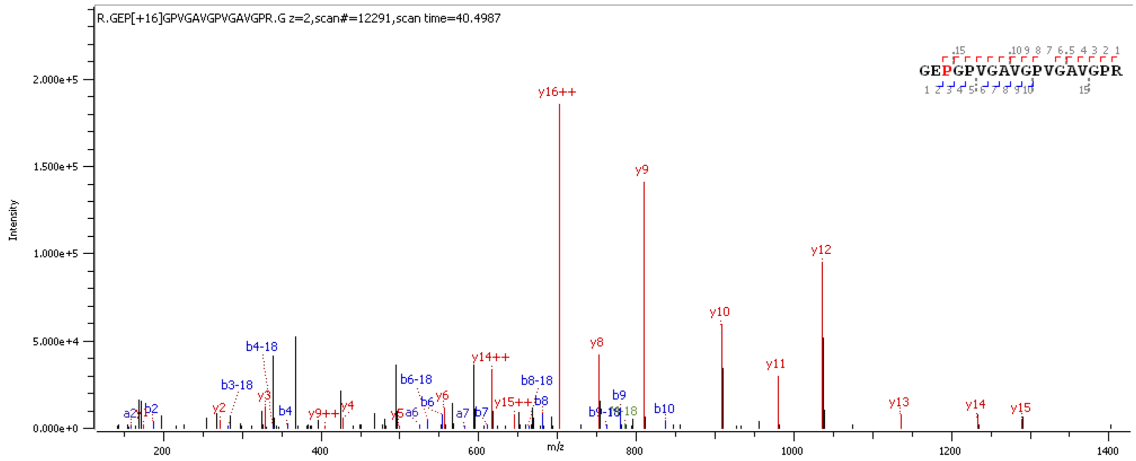

m/z
1590

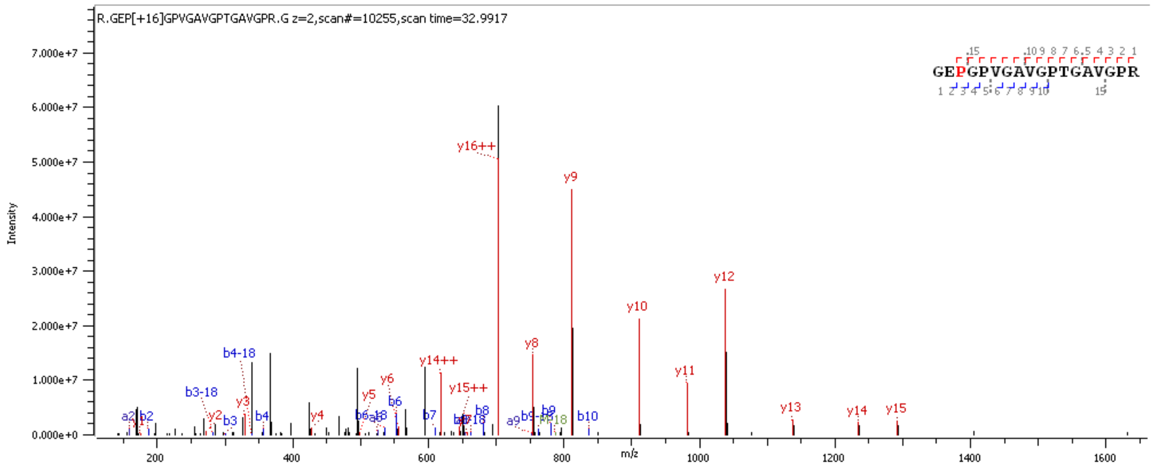

COL1α2 889 – 906 (novel marker)

Two version of the peptide with m/z 1560

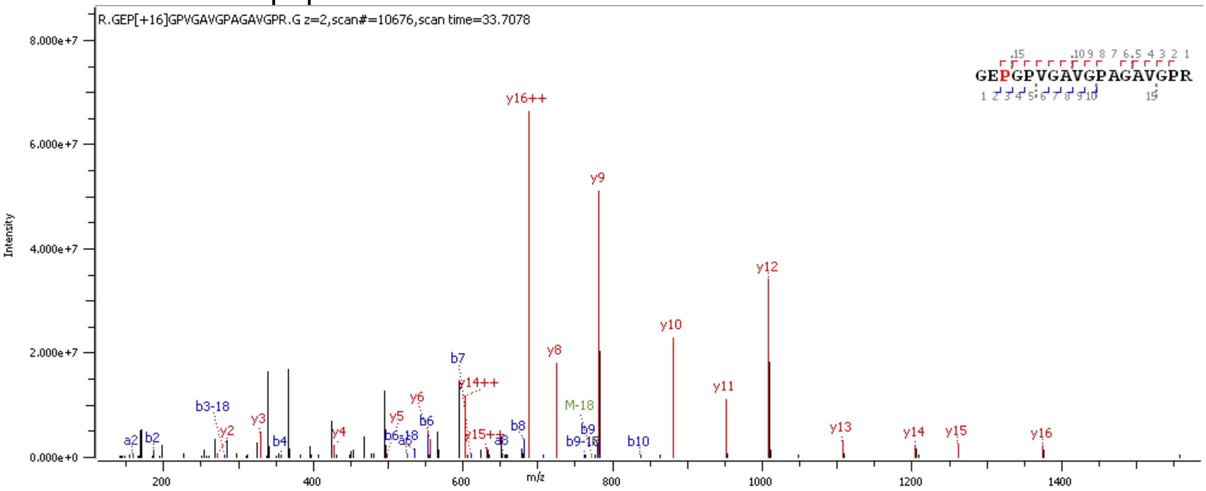

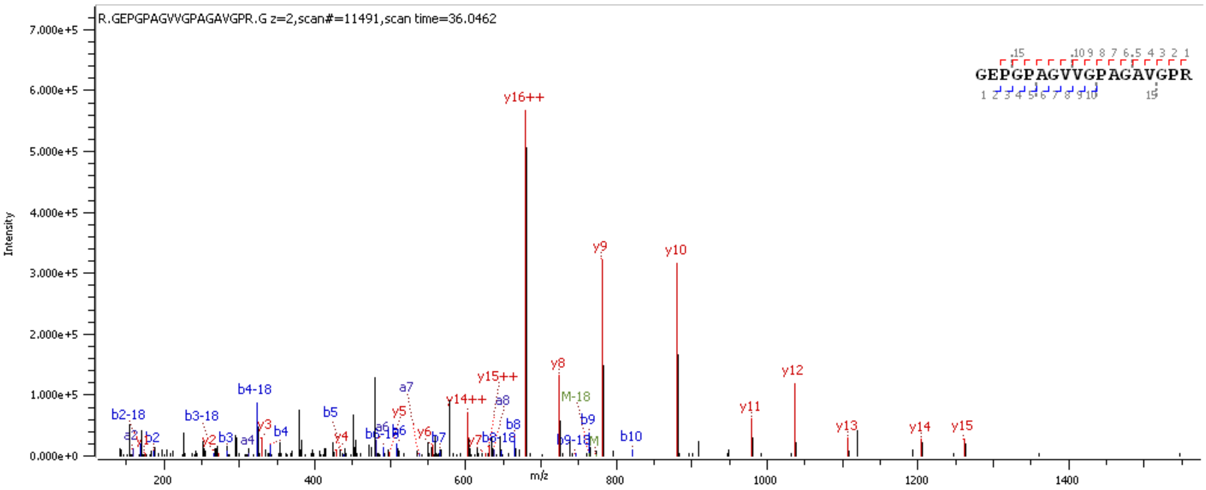

COL1α2 978 - 990 (A)

m/z 1150

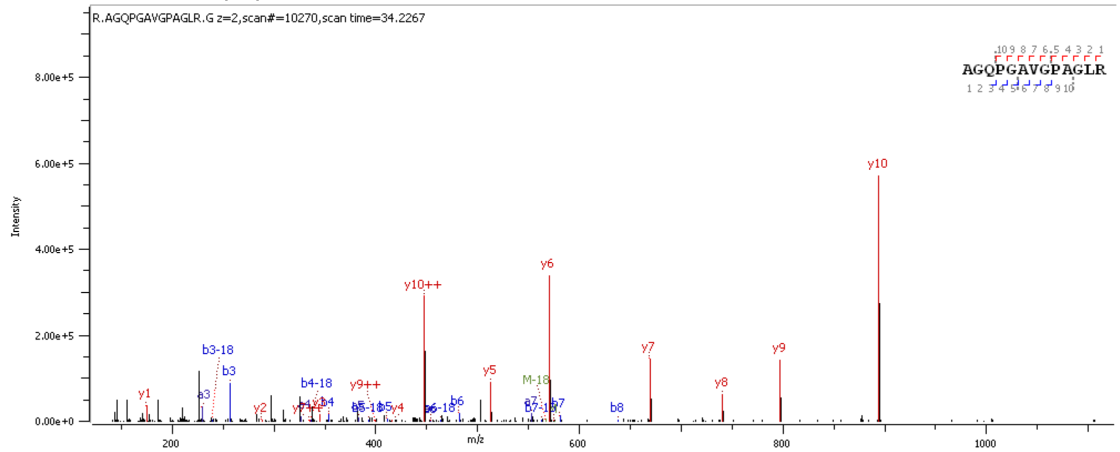

m/z 1166

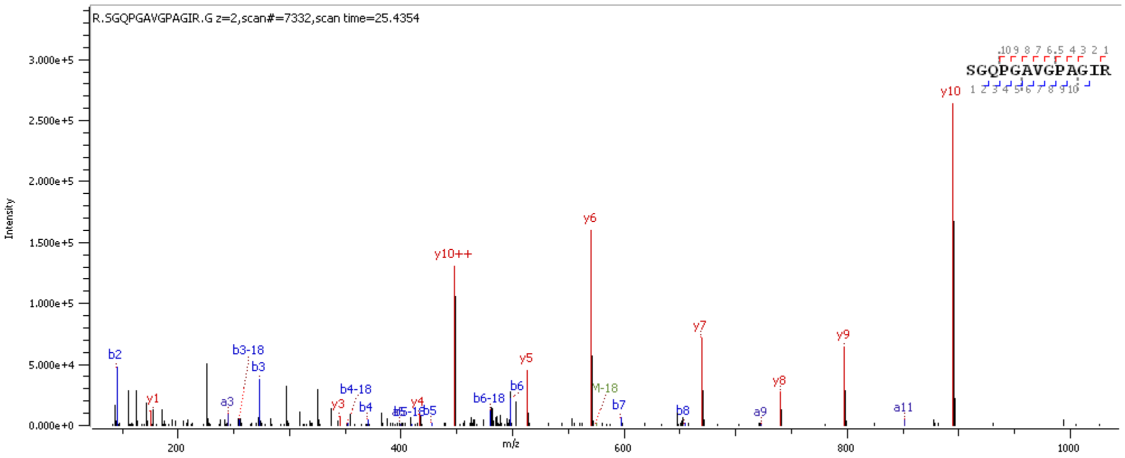

m/z 1180

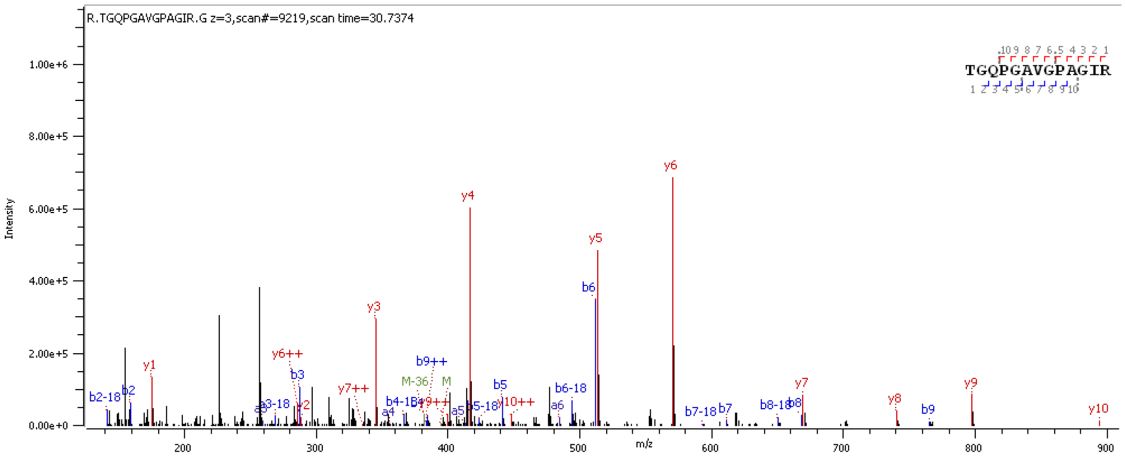

m/z 1192

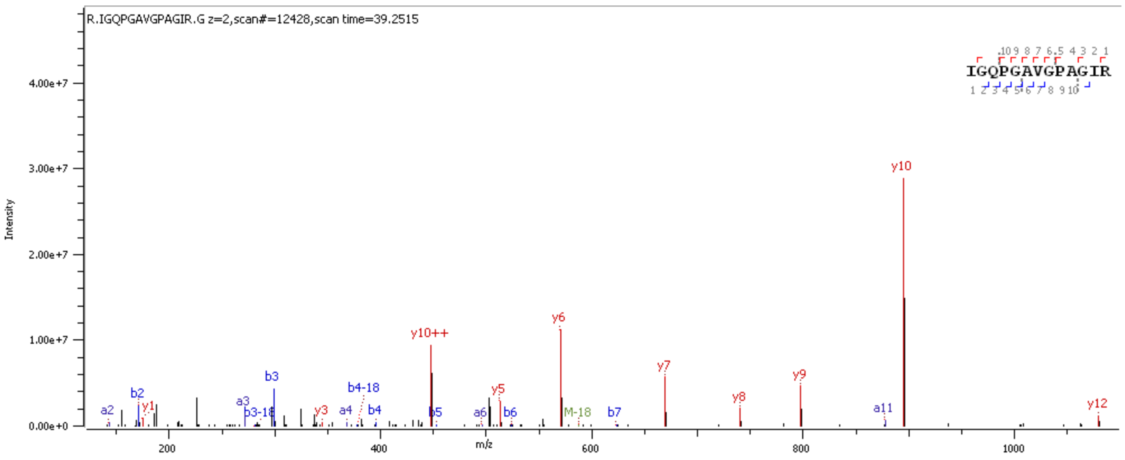

Supplement: S2 Fig — The following pages show the MS/MS spectra of the peptides at each of the diagnostic biomarkers presented in the manuscript. All images from Byonic. (PDF) [file pone.0251061.s002.pdf]
