# Supplementary material for: Distinguishing African bovids using Zooarchaeology by Mass Spectrometry (ZooMS): New peptide markers and insights into Iron Age economies in Zambia
Source: PLoS One. 2021 May 18;16(5):e0251061. doi: 10.1371/journal.pone.0251061 (PMC8130928; doi:10.1371/journal.pone.0251061)

S3 Fig. MS/MS Sequence Identification of the peptides at m/z 3017

In Bovidae the peak at m/z 3017 is composed of two peptides. One peptide is diagnostic between different species (COL1α2 757 – 789 or G). The other peptide is shared between all bovids and therefore non-diagnostic (COL1α2 89 – 130). This is why m/z 3017 should not be used for taxonomic identification. The diagnostic peptide is the only peptide which composes the MALDI peak with a different number of oxidations (m/z 3033) and therefore that is diagnostic. Images from Byonic.

COL1α2 757 – 789 (G)

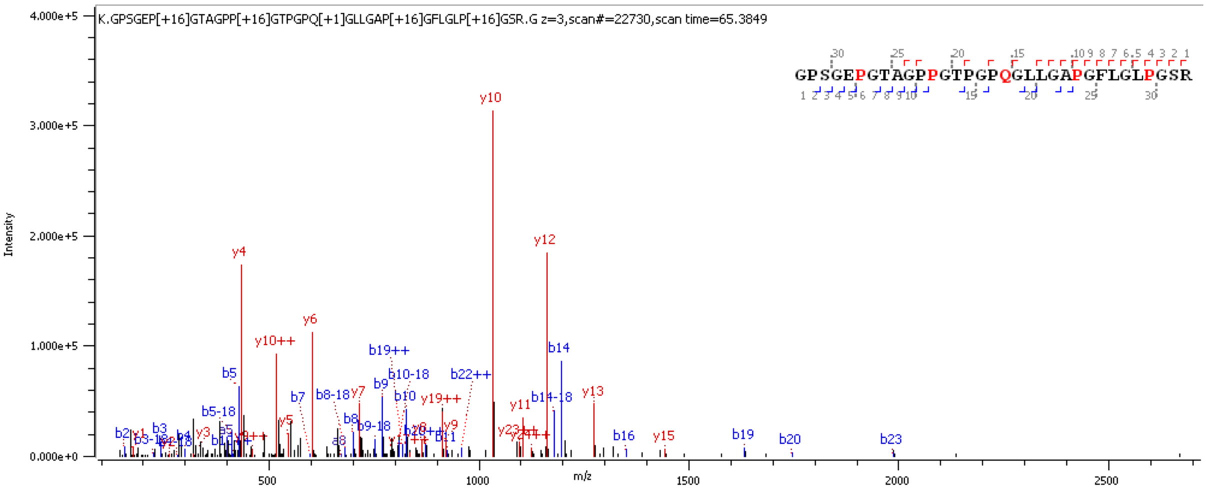

COL1α2 89 – 130

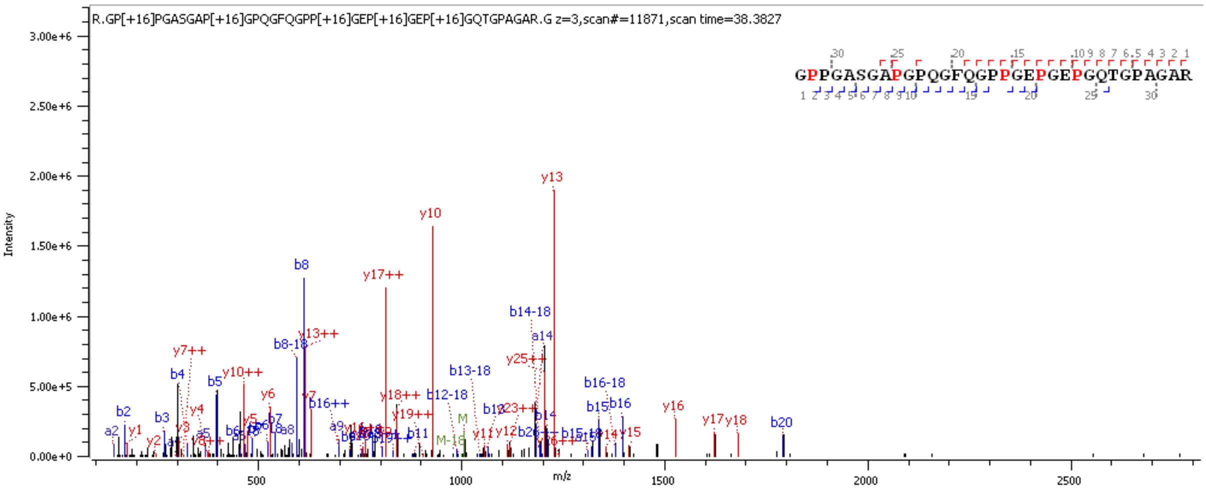

Supplement: S3 Fig — In Bovidae the peak at m/z 3017 is composed of two peptides. One peptide is diagnostic between different species (COL1α2 757–789 or G). The other peptide is shared between all bovids and therefore non-diagnostic (COL1α2 89–130). This is why m/z 3017 should not be used for taxonomic identification. The diagnostic peptide is the only peptide which composes the MALDI peak with a different number of oxidations (m/z 3033) and therefore that is diagnostic. Images from Byonic. (PDF) [file pone.0251061.s003.pdf]
