# Supplementary material for: Distinguishing African bovids using Zooarchaeology by Mass Spectrometry (ZooMS): New peptide markers and insights into Iron Age economies in Zambia
Source: PLoS One. 2021 May 18;16(5):e0251061. doi: 10.1371/journal.pone.0251061 (PMC8130928; doi:10.1371/journal.pone.0251061)
Supplement: S1 File — (PDF) [file pone.0251061.s009.pdf]

S1 File

Distinguishing African bovids using Zooarchaeology by Mass Spectrometry (ZooMS): New peptide markers and assessments of Iron Age economies in Zambia

The raw data has been uploaded to the following locations for open access:

MS/MS Data:	ProteomeXchange through MassIVE<br>Record Number: PXD020810<br>doi:10.25345/C5TJ3M password: reviewer
Reference ZooMS Spectra:	Zenodo<br>doi:10.5281/zenodo.3964709
Zambia ZooMS Spectra:	Zenodo<br>doi:10.5281/zenodo.3971142

Also uploaded with the Reference ZooMS Spectra to Zenodo under the same DOI are:

1. Aligned FASTA files for the mature peptides of col1α1 and col1α2 of all sequences used in the manuscript.
2. Two .gff files (col1α1 and col1α2) of the cattle sequence with the annotations for the locations of the mature peptide, helical region, and biomarkers presented in this paper.

Included in this Supporting Information PDF

1. Nomenclature of ZooMS markers
2. Note on *Neotragus pygmaeus*

Nomenclature of ZooMS markers

To avoid confusing marker nomenclature especially in the naming of new markers we employ the nomenclature method that is being presented in a paper currently under review. In order to ensure that the nomenclature method is understood all currently published markers are given with both the new nomenclature and their commonly used letter nomenclature. The new markers are named according to this convention.

The new nomenclature gives the names as the gene followed by the start and end positions of the peptide from the start position of the collagen triple helix. After this point there are no insertions or deletions making the system standardized across species. In COL1α1, the start of the helical region is GPMGPXGPR (where X is variable) at position 178 in the *Bos taurus* reference sequence (UniProt: P02453). In COL1α2, it is GPMGLMGPR, at position 89 in the *Bos taurus* reference sequence (UniProt: P024650). The following table shows the commonly used markers with their letter names and the new nomenclature.

Letter	Proposed Full Name
Cet1 / P1	COL1α1 508 - 519
F	COL1α1 586 - 618
Cet2 / P2	COL1α2 292 - 309
E	COL1α2 454 - 483
B	COL1α2 484 - 498
C	COL1α2 502 - 519
G	COL1α2 757 - 789
D	COL1α2 793 - 816
A	COL1α2 978 - 990

Note on *Neotragus pygmaeus*

The genetic sequence data obtained for *N. pygmaeus* had multiple unique single amino acid polymorphisms from the rest of the bovids including in several of the marker regions. We only reported markers where we could confirm the sequence using LC-MS/MS for at least one species. Therefore we did not report any of the markers for *N. pygmaeus* as we had no samples for analysis. More work will need to be done to identify and confirm markers for all of Neotragini, especially *N. pygmaeus*.
